# Improving disaggregation models of malaria incidence by ensembling non-linear models of prevalence

**DOI:** 10.1016/j.sste.2020.100357

**Published:** 2022-06

**Authors:** Tim C.D. Lucas, Anita K. Nandi, Suzanne H. Keddie, Elisabeth G. Chestnutt, Rosalind E. Howes, Susan F. Rumisha, Rohan Arambepola, Amelia Bertozzi-Villa, Andre Python, Tasmin L. Symons, Justin J. Millar, Punam Amratia, Penelope Hancock, Katherine E. Battle, Ewan Cameron, Peter W. Gething, Daniel J. Weiss

**Affiliations:** a Malaria Atlas Project, Big Data Institute, University of Oxford, Oxford, UK; b Institute for Disease Modeling, Bellevue, WA, USA; c Telethon Kids Institute, Perth Childrens Hospital, Perth, Australia; d Curtin University, Perth, Australia

**Keywords:** Spatial statistics, Disaggregation regression, Stacking, Surveillance data

## Abstract

Maps of disease burden are a core tool needed for the control and elimination of malaria. Reliable routine surveillance data of malaria incidence, typically aggregated to administrative units, is becoming more widely available. Disaggregation regression is an important model framework for estimating high resolution risk maps from aggregated data. However, the aggregation of incidence over large, heterogeneous areas means that these data are underpowered for estimating complex, non-linear models. In contrast, prevalence point-surveys are directly linked to local environmental conditions but are not common in many areas of the world. Here, we train multiple non-linear, machine learning models on *Plasmodium falciparum* prevalence point-surveys. We then ensemble the predictions from these machine learning models with a disaggregation regression model that uses aggregated malaria incidences as response data. We find that using a disaggregation regression model to combine predictions from machine learning models improves model accuracy relative to a baseline model.

## 1 Introduction

High-resolution maps of malaria risk are vital for control and elimination (Battle, Lucas, Nguyen, Howes, Nandi, Twohig, Pfeffer, Cameron, Rao, Casey, et al., 2019, Weiss, Lucas, Nguyen, Nandi, Bisanzio, Battle, Cameron, Twohig, Pfeffer, Rozier, et al., 2019). However, mapping malaria in lower burden countries presents new challenges as traditional mapping of prevalence from cluster-level surveys (Battle, Lucas, Nguyen, Howes, Nandi, Twohig, Pfeffer, Cameron, Rao, Casey, et al., 2019, Bhatt, Cameron, Flaxman, Weiss, Smith, Gething, 2017, Bhatt, Weiss, Cameron, Bisanzio, Mappin, Dalrymple, Battle, Moyes, Henry, Eckhoff, et al., 2015, Weiss, Lucas, Nguyen, Nandi, Bisanzio, Battle, Cameron, Twohig, Pfeffer, Rozier, et al., 2019) is often not effective for two reasons. Firstly, so few individuals are infected that most surveys will detect zero positives (Sturrock et al., 2016). Secondly, there is a lack of nationally representative prevalence surveys in low burden countries (Sturrock, Bennett, Midekisa, Gosling, Gething, Greenhouse, 2016, Sturrock, Cohen, Keil, Tatem, Le Menach, Ntshalintshali, Hsiang, Gosling, 2014). Routine surveillance data of malaria case counts, often aggregated over administrative regions defined by geographic polygons, is becoming more reliable and more widely available (Sturrock et al., 2016) and recent work has focussed on methods for estimating high-resolution malaria risk from these data (Johnson, Diggle, Giorgi, 2019, Law, Sejdinovic, Cameron, Lucas, Flaxman, Battle, Fukumizu, 2018, Li, Brown, Gesink, Rue, 2012, Sturrock, Cohen, Keil, Tatem, Le Menach, Ntshalintshali, Hsiang, Gosling, 2014, Taylor, Andrade-Pacheco, Sturrock, 2017, Wilson, Wakefield, 2018). However, the aggregation of cases over space means that the data may be spatially uninformative, especially if the case counts are aggregated over large or heterogeneous areas, because it is unclear where within the polygon, and in which environments, the cases occurred. This data is therefore often under-powered for fitting flexible, non-linear models as is required for accurate malaria maps (Bhatt, Cameron, Flaxman, Weiss, Smith, Gething, 2017, Bhatt, Weiss, Cameron, Bisanzio, Mappin, Dalrymple, Battle, Moyes, Henry, Eckhoff, et al., 2015). A method that combines prevalence point-surveys and aggregated surveillance data, and therefore leverages the strength of both, has great potential.

Here we propose a two-stage method. In the first stage we train a suite of machine learning models, using point-level, binomial prevalence data and environmental covariates. In the second stage we combine predictions from these models by using them as covariates in a polygon-level, disaggregation regression model that uses malaria incidence (aggregated to administrative units) as the response. Unlike joint likelihood models (Wang et al., 2018), this method does not combine both prevalence and incidence data within one model. Instead the aim is to use the prevalence data to find useful non-linear transformations of the environmental covariates which are then subsequently used in the disaggregation regression models.

Stacking, or stacked generalization, uses a second-stage model to combine predictions from a number of models by training the second-stage model using out-of-samples predictions from the first-stage models (Wolpert, 1992). The modelling scheme proposed here has similarities to stacking methods used for malaria mapping (Bhatt et al., 2017) and elsewhere (Breiman, 1996, Hao, Elith, Guillera-Arroita, Lahoz-Monfort, 2019, Sill, Takács, Mackey, Lin, Wolpert, 1992). However, as the response data in the machine learning models and the disaggregation regression models are on different scales (prevalence is a proportion while incidence is a rate) we cannot simply take a weighted average of the predictions from the machine learning models as in a standard stacking scheme (Hao, Elith, Guillera-Arroita, Lahoz-Monfort, 2019, Sill, Takács, Mackey, Lin). Instead the predictions need to be transformed to the incidence scale with a seperately fitted model (Cameron et al., 2015). Applications in other disease contexts have used a similar stacking scheme where data from vector or wild-animal host species are used to train models, the predictions from which are then used as covariates in a final model (Pigott, Golding, Mylne, Huang, Henry, Weiss, Brady, Kraemer, Smith, Moyes, et al., 2014, Shearer, Huang, Weiss, Wiebe, Gibson, Battle, Pigott, Brady, Putaporntip, Jongwutiwes, et al., 2016). In such applications we would always expect to need additional covariates as well as the modelled distributions of hosts or vectors. However, in the case examined here, both sets of data are direct measures of some aspect of malaria transmission rate, and therefore it is possible, though not guaranteed, that we would not need any further covariates.

Model stacking (Wolpert, 1992) has proven effective in many realms (Bhatt, Cameron, Flaxman, Weiss, Smith, Gething, 2017, Breiman, 1996, Hao, Elith, Guillera-Arroita, Lahoz-Monfort, 2019, Sill, Takács, Mackey, Lin). Stacking improves predictions by controlling bias and variance; as long as suitably diverse models are averaged, they will have different biases while high variance in models should be averaged out. This understanding of how stacking improves model performance indicates that diversity in models is important for stacking to be effective. Diversity in models is typically created in two ways: by using diverse training datasets (Breiman, 1996a) (as in Random Forests for example) and by using functionally different models (for example by averaging tree based models and neural networks) (Breiman, 1996b). One important trade-off in spatial modelling is whether to use local data (with a smaller sample size but that is likely to be representative of the area of study) or global data that have a larger sample size but a less close association with the areas of study. For the application of malaria mapping, we can think about diversity of training data in this context and expect that stacking separate models trained on local and global data will also increase the diversity of predictions in a useful way.

To test the effectiveness of the proposed approach we used data from four countries with relatively complete surveillance data: Madagascar, Colombia, Indonesia and Senegal. We focused our analysis on comparing the predictive performance of disaggregation regression when given different sets of covariates. Therefore we keep the structure of the disaggregation regression model the same and only vary the covariates provided to the model. In each country we fitted stage 1 machine learning models trained on prevalence data and raw environmental covariates. We made new covariates using predictions from these models. We then tested whether stage 2 disaggregation regression models with these new covariates performed better than a baseline disaggregation regression model that directly used the raw environmental covariates. We tested this approach using machine learning models trained on local prevalence data as well as models trained on a global prevalence dataset. While there was no consistently best model we found that, in most cases, the two stage method worked better than the single stage baseline disaggregation regression models. Using predictions from machine learning models, trained on local prevalence data as covariates improved the performance of disaggregation regression models relative to the disaggregation regression models that only used the raw environmental covariates. In contrast, using predictions from machine learning models trained on the global prevalence dataset rarely improved predictive performance.

## 2 Methods

### 2.1 Epidemiological data

We used two data sources that reflect *P. falciparum* malaria transmission; point-prevalence surveys and polygon-level, aggregated incidence data. We selected Madagascar, Colombia, Indonesia and Senegal as case examples as they all have fairly complete, publicly available, surveillance data at a finer geographical resolution than administrative level one (i.e. higher resolution than state or province). The prevalence survey data were extracted from the Malaria Atlas Project prevalence survey database using only data from 1990 onwards (Bhatt, Weiss, Cameron, Bisanzio, Mappin, Dalrymple, Battle, Moyes, Henry, Eckhoff, et al., 2015, Guerra, Hay, Lucioparedes, Gikandi, Tatem, Noor, Snow, 2007, Pfeffer, Lucas, May, Harris, Rozier, Twohig, Dalrymple, Guerra, Moyes, Thorn, et al., 2018). While the data covered a large time period, we did not model time explicitly as we are here focussed on spatial, rather than temporal modelling. Although we have not accounted for time in the models, as long as sampling in space is independent of time, the correct relationships should be recovered. The prevalence points were then standardised to an age range of 2–10 using the model from (Smith et al., 2007). This data was used as both a global dataset and as regional subsets. The global dataset contains 55,914 surveys in 44,842 distinct locations and represents samples from 5,687,304 individuals. As there were relatively few surveys in Colombia we used all points from South America (7,719 individuals from 522 locations) while for the other countries we used only data from that country (Madagascar: 89,381 individuals from 1505 locations. Indonesia: 1,512,888 individuals from 4778 locations. Senegal: 80,896 individuals from 1762 locations).

The polygon incidence data (i.e. malaria incidence aggregated to administrative units) were collected from government reports (Colombian National Institute of Health, Indonesia Ministry of Health, Rakotorahalahy, Senegal Ministry of Health) and standardised using methods defined in Cibulskis et al. (2011). This standardisation step accounts for missed cases due to lack of treatment seeking, missing case reports, and cases that sought medical attention outside the public health systems (Battle et al., 2016). For reports where cases were not reported at the species level, national estimates of the ratio between *P. falciparum* and *Plasmodium vivax* cases from the World Malaria Report were used to calculate *P. falciparum* only cases (World Health Organization, 2016). For incidence rates we divide by 1000 to give the Annual Parasite Index (API). To keep the analysis focused on spatial estimates we selected one year of surveillance data for each country. We used annual surveillance data from 2013 for Madagascar (110 districts), 2015 for Colombia (952 municipalities), 2013 for Indonesia (244 regencies and cities) and 2009 for Senegal (34 departments). These years were selected as they had the most complete data in each case.

Raster surfaces (i.e. population gridded to 5 × 5 km pixels) of population for the years 2005, 2010 and 2015, were created using data from WorldPop (Gaughan, Stevens, Linard, Jia, Tatem, 2013, Linard, Gilbert, Snow, Noor, Tatem, 2012, Sorichetta, Hornby, Stevens, Gaughan, Linard, Tatem, 2015) and from GPWv4 (NASA, 2018) where WorldPop did not have values. Population rasters for the remaining years were created by linear interpolation.

### 2.2 Raw environmental covariates

We considered a suite of environmental and anthropological covariates, at a resolution of approximately 5 × 5 kilometres that included the annual mean and log standard deviation of land surface temperature, enhanced vegetation index, malaria parasite temperature suitability index, elevation, tasseled cap wetness, log accessibility to cities and log night lights (Gething, Van Boeckel, Smith, Guerra, Patil, Snow, Hay, 2011, Weiss, Bhatt, Mappin, Van Boeckel, Smith, Hay, Gething, 2014, Weiss, Mappin, Dalrymple, Bhatt, Cameron, Hay, Gething, 2015, Weiss, Nelson, Gibson, Temperley, Peedell, Lieber, Hancher, Poyart, Belchior, Fullman, et al., 2018). All covariates were aligned in their native resolution (500 m or 1 km) and then aggregated to 5 km resolution, therefore there should be minimal effects from spatial misalignment. The covariates were standardised and centered to have a mean of zero and a standard deviation of one. We refer to this set of transformed variables as the raw environmental covariates (even though some of the covariates are anthropogenic rather than environmental) to distinguish them from other covariates created from predictions from stage 1 machine learning models. The raw environmental variables were used as covariates in the stage 1 machine learning models as well as being used directly as covariates in the baseline stage 2 disaggregation regression models.

### 2.3 Stage 1 machine learning models

For each country specific dataset and for the global dataset we fitted 5 stage 1 models via *caret* (Kuhn et al., 2017): elastic net (Zou and Hastie, 2012), Random Forest (Wright and Ziegler, 2015), projection pursuit regression (Friedman and Stuetzle, 1981), neural networks (Venables and Ripley, 2002) and boosted regression trees (gradient boosted models, subsequently GBM) (Ridgeway, 2017). These models were fitted to both the full malaria prevalence dataset and to the regional subsets of the data. Our response variable was prevalence and we weighted the data by sample size (i.e. the number of people tested for malaria in each survey). We used the raw environmental covariates described above as covariates in these machine learning models. This process can therefore be seen as creating non-linear transformations of the raw covariates that are hopefully better correlated with malaria incidence than the raw environmental covariates are. For each model we ran five-fold cross-validation to select hyperparameters using random search for Random Forest and boosted regression trees and grid search for the other models. Root mean square error (RMSE) was used to select the best performing model. We note that spatial or random cross-validation could have been used in this step. The choice is less critical than for the cross-validation scheme used to test model performance but using random cross-validation might select for hyperparameters giving more complex or flexible models. To an extent, when ensembling models, high variance is better than high bias as the variance gets averaged out (Breiman, 1996b).

**Fig. 1:**
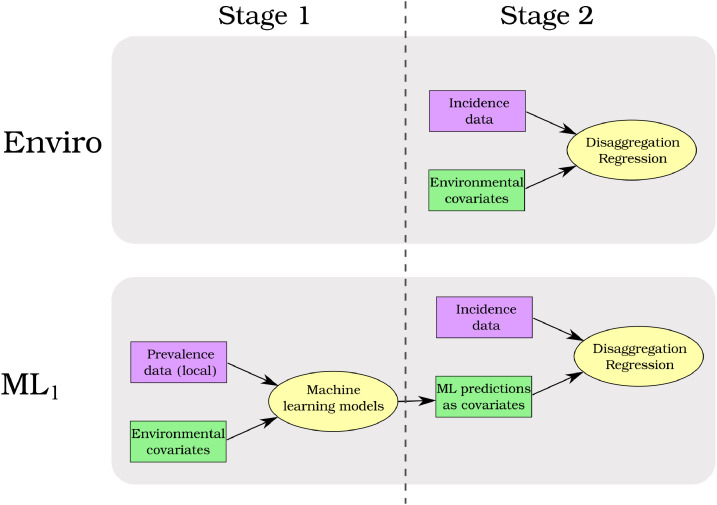
Schematic of the baseline disaggregation regression model (Enviro) and the two stage method (ML_l_). Models are shown in yellow ovals, malaria data is shown in purple rectangles and covariates are shown in green rectangles. The baseline model (Enviro) uses aggregated incidence data and raw environmental covarates in a disaggregation regression model. In the two stage method (ML_l_), new covariates are created in stage 1 by training machine learning models on prevalence data. Predictions from these machine learning models are used as covariates in the stage 2 disaggregation regression. Only one of the two stage models (ML_l_) is shown for simplicity. If ML_g_ was included as well for example, it would look the same as ML_l_ except that the prevalence data (pink box in stage 1) would have the global database of prevalence surveys. (For interpretation of the references to colour in this figure legend, the reader is referred to the web version of this article.)

Predictions from these models were then made across each country respectively. These predictions were empirical logit transformed so that they were on the linear predictor scale of the disaggregation regression model. An empirical logit was used rather than a standard logit as there were many predictions of exactly zero. These predicted surfaces were subsequently used as covariates in the stage 2 disaggregation regression models (Fig. 1). Plots of the correlation matrices for all covariates can be seen in S46S49. The correlation between covariates varied from country to country. In general there is little correlation between global and local machine learning predictions. Accessibility is often strongly correlated with predictions from the machine learning models. In Senegal there is a lot of correlation between variables. However, we note that collinearity between variables is not as problematic in a predictive context as it is when interpreting regression coefficients is the aim of the analysis, though it is still potentially a waste of degrees of freedom. The experiments that follow in this paper assess the performance of disaggregation regression models when using the predictions from these machine learning models as covariates as compared to a baseline disaggregation regression model using only the raw environmental covariates. See the supplementary material for plots of the grid search hyperparameter performance, out-of-sample scatter plots and plots of the predicted surfaces.

### 2.4 Stage 2 disaggregation regression

The model fitted to aggregated incidence data was a disaggregation regression model (Law, Sejdinovic, Cameron, Lucas, Flaxman, Battle, Fukumizu, 2018, Li, Brown, Gesink, Rue, 2012, Sturrock, Cohen, Keil, Tatem, Le Menach, Ntshalintshali, Hsiang, Gosling, 2014, Taylor, Andrade-Pacheco, Sturrock, 2017, Wilson, Wakefield, 2018). The models were implemented and fitted using Template Model Builder (Kristensen et al., 2016) in R (R Core Team, 2018) and we note that these models cannot be fitted using INLA (Lindgren and Rue, 2015) as we are not using a linear link function. This model is defined by a likelihood at the level of the polygon with covariates and a spatial random field at the pixel-level. Values at the polygon-level are given the subscript *a* while pixel level values are indexed with *b*.

The aggregated incidence count data, *y_a_* is given a Poisson likelihood

$$y_{a}∼\mathrm{Pois}\left(i_{a}{\text{pop}}_{a}\right)$$

where *i_a_* is the estimated polygon incidence rate and pop_a_ is the observed polygon population-at-risk. This polygon-level likelihood is linked to the pixel-level incidence and prevalence by

$$i_{a}=\frac{\sum \left(i_{b}{\mathrm{pop}}_{b}\right)}{\sum {\mathrm{pop}}_{b}}$$

$$i_{b}=2.616p_{b}-3.596p_{b}^{2}+1.594p_{b}^{3}$$

where the polynomial is a function from a previously published model (Cameron et al., 2015). The fact that the model is explicit about the relationship between prevalence and incidence has two advantages. Firstly, predictions of prevalence can be easily made directly from the linear predictor of the model. Secondly, it means that the logit-transformed predictions from the machine learning models are correctly scaled. The linear predictor of the model is related to prevalence by a typical logit link function and includes: an intercept, *β*_0_; covariates, *X*, with a vector of regression parameters, *β*; a spatial, Gaussian, random field, *u_b_*(*ρ, σ_u_*); and an *iid* random effect, *v_a_*(*σ_v_*).

$$p_{b}={\mathrm{logit}}^{-1}\left(\beta_{0}+\beta X+u_{b}\left(\rho ,\sigma_{u}\right)+v_{a}\left(\sigma_{v}\right)\right)$$

The Gaussian spatial effect has a Matérn covariance function and two hyperparameters: *ρ*, the nominal range (beyond which correlation is  < 0.1) and *σ_u_*, the marginal standard deviation. The *iid* random effect models both unobserved explanatory factors and extra-Poisson sampling error. As described in more detail below we do not vary the structure of this model in the methodological comparison. The matrix of covariates, *X*, it the only component that varies and is made up of various combinations of raw environmental covariates and predictions from the machine learning models.

Finally, we complete the model by setting priors on the parameters *β*_0_, *β, ρ* and *σ_u_* and *σ_v_*. We assigned *ρ* and *σ_u_* a joint penalised complexity prior (Fuglstad et al., 2018) such that P(ρ<1)=0.0001 (except for Indonesia where we set P(ρ<3)=0.0001 due to its much larger size) and $P(\sigma_{u}>1)=0.0001$. This prior encoded our *a priori* preference for a simpler, smoother random field. We set this prior such that the random field could explain most of the range of the data if required.

We assigned *σ_v_* a penalised complexity prior (Simpson et al., 2017) such that $P(\sigma_{v}>0.05)=0.0001$. This was based on a comparison of the variance of Poisson random variables, with rates given by the number of polygon-level cases observed, and an independently derived upper and lower bound for the case counts using the approach defined in (Cibulskis et al., 2011). We found that an *iid* effect with a standard deviation of 0.05 would be able to account for the discrepancy between the assumed Poisson error and the independently derived error.

Finally, we set different priors on the regression coefficients depending on which covariates were used. When raw environmental covariates or a mix of raw environmental covariates and predictions from machine learning models were used we set the prior to be weakly regularising, $\beta_{i}∼\mathrm{Norm}\left(0,0.4\right),$ such that it was unlikely that any single covariate could explain the full range of the response data. When only machine learning model predictions were used we set $\beta_{i}∼\mathrm{Norm}\left(\frac{1}{M},0.4\right)$ where *M* is the number of machine learning models being used. This prior sets our *a priori* expectation that all the machine learning prediction models are positively and equally correlated with incidence i.e. this prior encodes standard model averaging. It is important to note that this setup does not constitute true stacking in which we would enforce $\sum_{i}\beta_{i}=1$ (Bhatt et al., 2017). In a preliminary analysis we tested the case where we force *β_i_* > 0 which in practice is largely the same as the $\sum_{i}\beta_{i}=1$ (Breiman, 1996b) but allows a small amount of flexibility to handle mispecification in p2i. This analysis did not show any benefits to this approach so it was not considered further.

### 2.5 Experiments

All experiments involve comparing predictive performance of stage 2 disaggregation regression models when different combinations of covariates are used. After model fitting we made predictions over the study areas and reaggregated back to the administrative level of the surveillance data. Our primary performance metric was correlation between observed and aggregated predictions. We also examined the calibration of models by calculating the proportion of held out data that were within their 80% credible intervals. We used the raw environmental covariates, centered and standardised, as a baseline stage 2 model (Fig. 1). We subsequently refer to this model as Enviro. We then performed two experiments. In the first (experiment 1) we tested whether using predictions from stage 1 machine learning models trained on local (i.e. within-country) prevalence data improved predictions (Fig. 1). In the second (experiment 2) we tested whether using predictions from stage 1 machine learning models trained on global prevalence data improved predictions.

In experiment 1 we compared the baseline to two models that used locally trained machine learning models (Table 1). Firstly, we combined the predictions from the machine learning models and the environmental covariates in one model (subsequently called Enviro + ML_l_). In this model therefore, the environmental covariates are effectively used twice, once in their raw form and once in transformed form (i.e. the predictions from the machine learning models). Secondly, we used only the predictions from the machine learning models (Fig. 1) trained on local data (subsequantly called ML_l_). As the environmental covariates are used as covariates in the machine learning models, this model is still ultimately driven by the values of the raw environmental covariates. However, in this model we are only using the environmental covariates in the transformed space learned by the machine learning models.

**Table 1:** Summary of stage 2 models and the experiments they are grouped into. All models are disaggregation regression models fitted to aggregated incidence data. The only difference between the models is which covariates are being used. Environmental covariates includes the full set of eight variables. Local ML covariates are the predictions from stage 1 machine learning models, trained with local prevalence data and the eight environmental covariates as inputs. The local prevalence datasets are data from within each country except for Colombia where local prevalence refers to South American data. Global ML covariates are the predictions from stage 1 machine learning models, trained with global prevalence data (i.e. the full prevalence database) and the eight environmental covariates as inputs. Experiment 1 tests whether including Local ML covariates improves predictive performance while Experiment 2 tests whether including Global ML covariates improves performance.

Model	Environmental covariates	Local ML covariates	Global ML covariates
**Experiment 1**			
Enviro	✓		
Enviro + ML_l_	✓	✓	
ML_l_		✓	
**Experiment 2**			
Enviro	✓		
ML_g_			✓
ML_l_ + ML_g_		✓	✓

In experiment 2 we compared the baseline model to two models that used predictions from machine learning models trained on the global dataset of prevalence surveys (Table 1). In the first, we used only predictions from the machine learning models trained on the global data (ML_g_). In the second, we combined predictions from the machine learning models trained on regional data and predictions from the machine learning models trained on global data (ML_l_ + ML_g_).

In each experiment we used two cross-validation schemes. In the first, polygon incidence data was randomly split into six cross-validation folds. In the second, polygon incidence data was split spatially into k folds (via k-means clustering on the polygon centroids). We set k as 3 for Madagascar and Colombia. Due to its large size we set k as 7 for Indonesia. Due to the small sample size, we set k as 5 for Senegal. This spatial cross-validation scheme is testing the ability of the different models to make predictions far from data where the spatial random field is not informative.

## 3 Results

Table 2 gives the correlation between observed and held out data (under random and spatial cross-validation) for experiment 1 (models Enviro, ML_l_ and Enviro + ML_l_). Many of the differences in performance are rather marginal. Enviro was the best performing model in two cases (random cross-validation in Madagascar and Senegal). In one case, Enviro perfomed equally well as another model; under spatial cross-validation in Senegal Enviro and Enviro + ML_l_ performed equally well. Of the remaining five cases, in two cases ML_l_ performed best and in three cases Enviro + ML_l_ performed best. The greatest benefits to using prediction from machine learning models instead of, or in combination with, the raw environmental variables occured under spatial cross-validation and in the cases when the Enviro model was particularly poor.

**Table 2:** Pearson correlations between observed and predicted values for experiment 1.

Country	Cross-validation	Enviro	Enviro + ML_l_	ML_l_
Colombia	Random	0.59	**0.61**	0.59
Colombia	Spatial	0.12	0.25	**0.33**
Indonesia	Random	0.52	**0.59**	0.48
Indonesia	Spatial	0.45	**0.51**	0.44
Madagascar	Random	**0.70**	0.69	0.68
Madagascar	Spatial	0.22	0.18	**0.55**
Senegal	Random	**0.58**	0.57	0.51
Senegal	Spatial	0.63	0.63	0.51

**Fig. 2:**
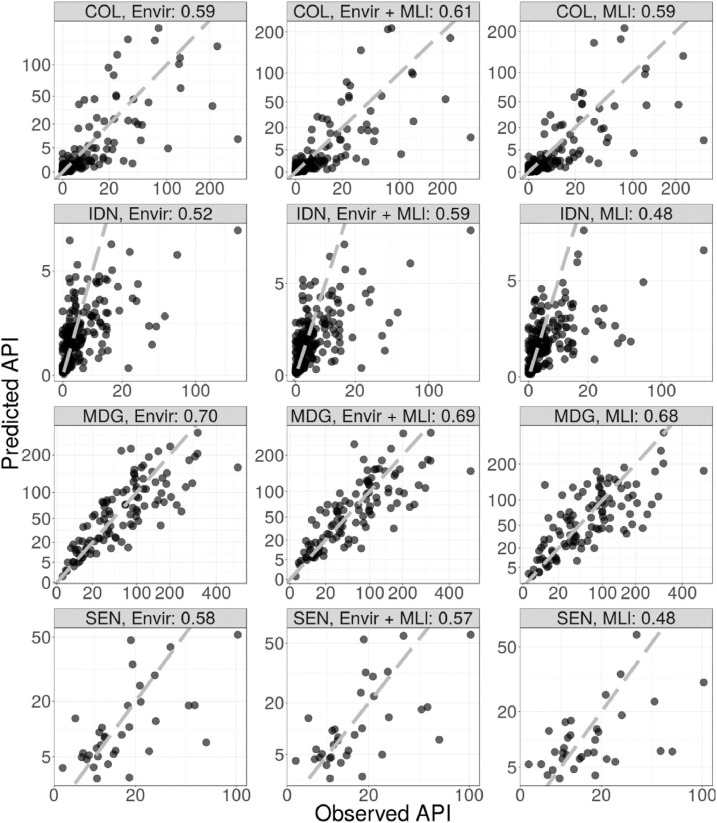
Observed data against predictions for random cross-validation hold-out samples on a square root transformed scale. There are 12 cases composed of 4 countries (COL:, Colombia, IDN: Indonesia, MDG: Madagascar, SEN: Senegal) and three sets of covariates (Envir: raw environmental covariates only, Enviro + ML_l_: raw environmental covariates and machine learning covariates trained on local prevalence data combined, ML_l_: Machine learning models trained on local prevalence data only.

**Fig. 3:**
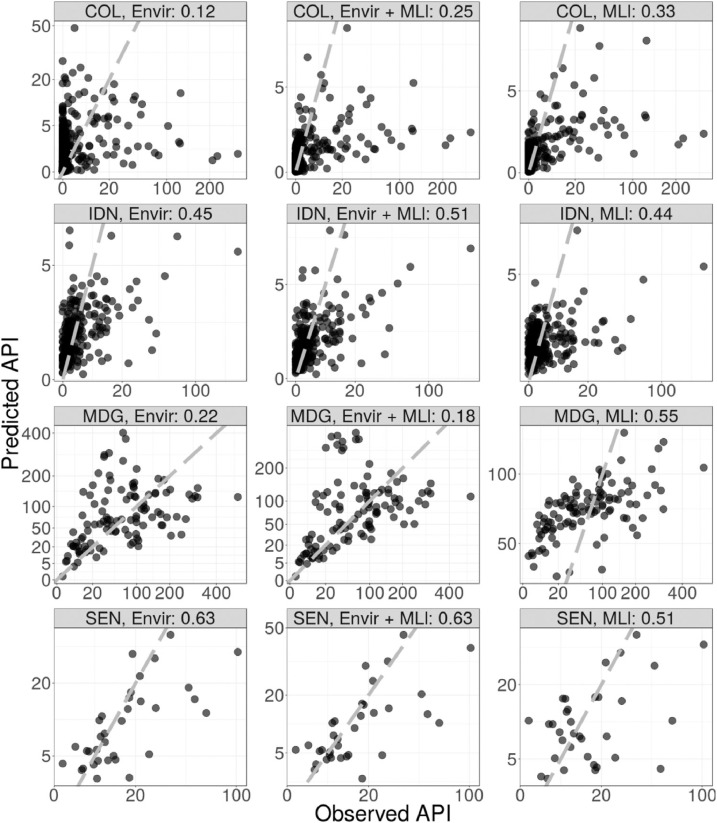
Observed data against predictions for spatial cross-validation hold-out samples on a square root transformed scale. There are 12 cases composed of 4 countries (COL:, Colombia, IDN: Indonesia, MDG: Madagascar, SEN: Senegal) and three sets of covariates (Envir: raw environmental covariates only, Enviro + ML_l_: raw environmental covariates and machine learning covariates trained on local prevalence data combined, ML_l_: Machine learning models trained on local prevalence data only.

**Fig. 4:**
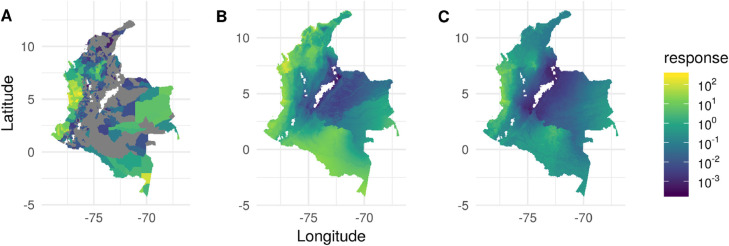
A) Observed data for Colombia (grey for zero incidence). B) Out-of-sample predictions for the spatial cross-validation, environmental covariates only model. C) Out-of-sample predictions for the spatial cross-validation, local machine learning only model. For each cross-validation fold, predictions are made for the held out data which are then combined to make a single surface.

Fig. 2 shows scatter plots of the model performance under random cross-validation for experiment 1 while Fig. 3 shows scatter plots of the model performance under spatial cross-validation. It can be seen that without environmental covariates, the models in Madagascar fail to predict very high or very low values correctly. Fig. 4 shows the input data and spatial out-of-sample predictions of the Enviro model and ML_l_ model in Colombia.

Table 3 gives the correlation between observed and held out data (under random and spatial cross-validation) for experiment 2 (models Enviro, ML_g_ and ML_l_ + ML_g_). In six cases, Enviro was the best or tied best performing model. The ML_g_ model was never the best performing model and only outperforms Enviro in one case (spatial cross-validation in Madagascar). In two cases ML_l_ + ML_g_ was the best performing model (spatial Colombia and spatial Madagascar). Comparing across Tables 2 and 3 we can see that ML_g_ outperforms ML_l_ once (spatial cross-validation in Senegal). In only two cases (Spatial Senegal and Spatial Indonesia) did ML_l_ + ML_g_ outperform ML_l_.

**Table 3:** Pearson correlations between observed and predicted values for experiment 2.

Country	Cross-validation	Enviro	ML_g_	ML_l_ + ML_g_
Colombia	Random	**0.59**	0.55	0.58
Colombia	Spatial	0.12	0.12	**0.33**
Indonesia	Random	**0.52**	0.32	0.46
Indonesia	Spatial	0.45	0.41	0.45
Madagascar	Random	**0.70**	0.67	0.68
Madagascar	Spatial	0.22	0.51	**0.55**
Senegal	Random	**0.58**	0.50	0.49
Senegal	Spatial	**0.63**	0.55	0.52

Table 4 shows the out-of-sample coverage of the 80% credible intervals for all models, countries and cross-validation schemes. The coverage in Colombia was very poor with no models achieving a coverage above 0.4. The coverage was acceptable in the other three countries with most values lying between 0.7 and 0.9. Overall there was a general tendency for models to be slightly over confident.

**Table 4:** Coverage of 80% credible intervals. Values outside 0.7–0.9 are shown in bold.

Country	CV	Enviro	ML_l_	Enviro + ML_l_	ML_g_	ML_l_ + ML_g_
Colombia	Random	**0.28**	**0.28**	**0.29**	**0.28**	**0.30**
Colombia	Spatial	**0.30**	**0.33**	**0.33**	**0.33**	**0.34**
Indonesia	Random	0.80	0.81	0.78	0.79	0.77
Indonesia	Spatial	0.80	0.78	0.78	0.76	0.75
Madagascar	Random	0.80	0.77	0.75	0.77	0.76
Madagascar	Spatial	**0.65**	0.74	0.70	0.75	0.76
Senegal	Random	0.79	0.79	0.79	0.79	0.82
Senegal	Spatial	0.85	**0.91**	0.85	**0.94**	0.85

We can examine the relationship between the RMSE of the machine learning models to their fitted regression coefficients (weights). These values are given in Table 5. In all five sets of machine learning models (four sets trained on local data and one set trained on global data), Random Forest performs the best. We have not forced *β_i_* > 0 but we have set the priors for these coefficients with a positive mean. In nearly all cases the fitted values are positive. If the prevalence data and incidence data are not biased relative to each other we would expect the models with the lowest RMSE to also have the biggest regression coefficient. This occurs in three cases where Random Forest has the lowest RMSE and the biggest coefficient. In a further two cases, Random Forest has the lowest RMSE but GBM has the highest coefficient. The predictions from Random Forest and GBM are highly correlated in part because they both perform well and in part because they are both tree based models. Finally, it can be seen that the relationship between RMSE and regression coefficients was much weaker in Indonesia. For the models trained on local data a neural network has the highest fitted coefficient while for the models trained on global data an elastic net has the highest fitted coefficient.

**Table 5:** Machine learning model results and fitted parameters (i.e. model weights) of the machine learning predictions only models (i.e. ML_l_ local only and ML_g_ global only). For each dataset (the country and whether the data was local or global) the best model (lowest RMSE) is shown in bold. Similary, the largest coefficient within each disaggregation model is shown in bold.

Country	Model	RMSE_l_	*β_l_*	RMSE_g_	*β_g_*
Colombia	RF	**0.068**	0.625	**0.169**	0.180
Colombia	GBM	0.073	**0.952**	0.178	-0.218
Colombia	enet	0.070	0.219	0.233	0.183
Colombia	nnet	0.070	0.129	0.220	0.527
Colombia	ppr	0.070	0.667	0.205	**0.546**
Indonesia	RF	**0.081**	0.447	**0.169**	0.178
Indonesia	GBM	0.085	0.357	0.178	0.289
Indonesia	enet	0.091	0.303	0.233	**0.526**
Indonesia	nnet	0.089	**0.506**	0.220	0.316
Indonesia	ppr	0.089	0.364	0.205	0.089
Madagascar	RF	**0.100**	0.538	**0.169**	**0.529**
Madagascar	GBM	0.105	**0.570**	0.178	0.432
Madagascar	enet	0.116	0.301	0.233	0.262
Madagascar	nnet	0.113	0.033	0.220	0.364
Madagascar	ppr	0.109	0.469	0.205	0.403
Senegal	RF	**0.092**	**0.339**	**0.169**	**0.425**
Senegal	GBM	0.099	0.261	0.178	0.408
Senegal	enet	0.103	0.344	0.233	0.205
Senegal	nnet	0.099	0.254	0.220	0.190
Senegal	ppr	0.098	0.268	0.205	0.126

## 4 Discussion

We have studied the predictive performance of disaggregation regression of malaria incidence when provided with different sets of covariates. In experiment 1 we compared a baseline model that used only raw environmental covariates (Enviro) to two models that used predictions from machine learning models trained on local prevalence points (ML_l_ and Enviro + ML_l_). Overall, experiment 1 suggests that the predictions from the disaggregation models were better when using covariates created using predictions from machine learning models (trained on local prevalence points) than when using raw environmental covariates. This increased performance comes despite the prevalence data being on a different scale (a proportion instead of a rate) and being measurements of a different aspects of malaria transmission (prevalence rather than incidence) as well as the fact that the model we have used to translate between the two scales is imperfect. However, there was no clear best model between ML_l_ and Enviro + ML_l_. Therefore, when using these methods, both of these models should be fitted and the model with the best predictive performance for a given dataset selected.

Furthermore, many of the performance improvements are rather marginal. However, in a few cases such as Colombia and Madagascar under spatial cross-validation the performance boost is large. For example, using just environmental covariates in Madagascar under spatial cross-validation gives a correlation between observed and predicted data of 0.22. Such a model would be unusable for any applied or policy work. In contrast, using the predictions from the machine learning models trained on local data gives a correlation value of 0.55, which while still relatively poor is possibly a useful model.

It is also of note that in two cases, the simplest model (Enviro) performed the best. Given that Enviro + ML_l_ includes the same covariates (with some extra) this is likely explained by increased variance. The model is trying to estimate a number of regression parameters, a random field and hyperparameters so removing extraneous covariates should help parameter estimation. This is particularly true in Senegal which only has 34 datapoints and higher correlation between covariates (S49). Future work should consider joint models which use the prevalence and incidence data as response data so that the disaggregation has more degrees of freedom. This would hopefully allow the accurate estimation of more regression parameters The prevalence to incidence relationship used in this paper could be used to link the two data types. Furthermore, if the parameters in the prevalence incidence relationship were treated as unknown parameters, with informative priors, this would allow three data to inform the relationship and for the uncertainty in the original model to be propagated properly.

As expected, the model performance was generally worse under spatial cross-validation than under random cross-validation. This implies that the models are still relying heavily on the spatial Gaussian random field. Furthermore, the difference between random and spatial cross-validation is usually bigger than the difference between different models. This suggests that better data coverage is more important than which specific model is used and that the models are still relying quite strongly on the random field.

Overall, the models fitted using predictions from machine learning models trained on the global database of prevalence point-surveys were worse than those using either environmental data alone or than those using predictions from machine learning models trained on local data. This in itself is not particularly surprising. However, it does indicate that continental scale models should consider using a mosaic of locally trained machine learning models for example. What is more surprising is that using both the ML_l_ and ML_g_ covariates together did not improve performance, especially considering the relatively low correlation between the local and global predictions (Figure S46-49). Given that this approach of creating diverse predictions is a core element of stacking methodology we would expect this set of covariates to perform as well or better than just the ML_l_ covariates.

We have demonstrated that using prevalence data alongside aggregated incidence data can improve predictive performance. Unlike the maps presented by Weiss et al. (2019), the estimates presented in this paper are intended for methodological research, not directly for policy decisions. However, they do further demonstrate that aggregated incidence data can be used to create high resolution estimates of malaria incidence. Furthermore, in countries such as Senegal, the incidence data is much coarser than intervention implementation units, so disaggregated maps like the ones here have clear policy uses. It is important, however, to note that we are using out-of-sample prediction of aggregated polygon data as our performance metric while the true target for prediction is the high resolution risk surface. In rare cases there is both aggregate and unaggregated data available so that the high resolution accuracy can be tested as well as the aggregate level performance (Sturrock et al., 2014). However, this case is rare. Some simulation studies have been performed (Johnson, Diggle, Giorgi, 2019, Law, Sejdinovic, Cameron, Lucas, Flaxman, Battle, Fukumizu, 2018, Wilson, Wakefield, 2018) but these are often with few covariates and on correctly specified models. This is an important area for future research.

While the approach presented here is related to stacking, it differs in that we have not constrained the regression parameters to be positive nor included a sum-to-one constraint, i.e. the result is not simply a weighted average of the level zero model predictions. We did not include these constraints because the first stage and second stage models were trained on response data on different scales. However, given our priors, nearly all the fitted regression coefficients in models with only machine learning predictions were positive. Therefore, in practice these models are working in a way similar to standard stacking. However, the coefficients certainly do not sum to one, and fitted intercepts are negative to account for this.

One drawback of using predictions from machine learnings models as covariates is that the uncertainties in the predictions are not propagated properly through to the final predictions. This could be handled with an appropriate error model (Richardson and Gilks, 1993). Where the machine learning models explicitly provide estimates of uncertainty, these could be used to inform such models. In the absence of such individual-prediction estimates of uncertainty, the cross-validation error could instead be used to inform priors for error models. It is however worth noting that the environmental covariates used here are also modelled and therefore similar care would ideally be taken in characterising the uncertainty in their values.

## 5 Conclusions

Overall we find that including predictions from machine learning models trained on prevalence point-surveys can improve the performance of disaggregation regression models for malaria incidence relative to using raw environmental covariates. This extra modelling step can be seen as finding useful, non-linear transformations of the raw environmental covariates. This view is important for understanding how the model will predict in areas with no incidence data; in this situation the data cannot inform the Gaussian random field and predictions are driven by this non-linear transformation of the raw environmental covariates. Training the machine learning models on local data (i.e. prevalence data from the same country or region as the incidence data) shows much better performance than when training the machine learning models on the full, global, dataset of prevalence point-surveys. More countries, particularly those with medium or low malaria burdens, are providing timely and accurate routine surveillance data of malaria cases. Therefore it may be expected that disaggregation regression may become more popular and operationally relevant. Indeed, methods similar to ML_g_ are already being used for global mapping in malaria (Battle, Lucas, Nguyen, Howes, Nandi, Twohig, Pfeffer, Cameron, Rao, Casey, et al., 2019, Weiss, Lucas, Nguyen, Nandi, Bisanzio, Battle, Cameron, Twohig, Pfeffer, Rozier, et al., 2019) though the results here suggest that future global mapping efforts should allow local prevalence data to have a stronger influence on the estimates. We have here presented a method for improving the predictive performance of these models by using ancillary, prevalence point-survey data.

## Appendix A Supplementary materials

Supplementary Data S1 Supplementary Raw Research Data. This is open data under the CC BY license http://creativecommons.org/licenses/by/4.0/ Supplementary Data S1

## References

[bib0001] Battle K.E., Bisanzio D., Gibson H.S., Bhatt S., Cameron E., Weiss D.J., Mappin B., Dalrymple U., Howes R.E., Hay S.I. (2016). Treatment-seeking rates in malaria endemic countries. Malar. J..

[bib0002] Battle K.E., Lucas T.C.D., Nguyen M., Howes R.E., Nandi A.K., Twohig K.A., Pfeffer D.A., Cameron E., Rao P.C., Casey D. (2019). Mapping the global endemicity and clinical burden of *Plasmodium vivax*, 2000–17: a spatial and temporal modelling study. Lancet.

[bib0003] Bhatt S., Cameron E., Flaxman S.R., Weiss D.J., Smith D.L., Gething P.W. (2017). Improved prediction accuracy for disease risk mapping using Gaussian process stacked generalization. J. R. Soc. Interface.

[bib0004] Bhatt S., Weiss D., Cameron E., Bisanzio D., Mappin B., Dalrymple U., Battle K., Moyes C., Henry A., Eckhoff P. (2015). The effect of malaria control on *Plasmodium falciparum* in Africa between 2000 and 2015. Nature.

[bib0005] Breiman L. (1996). Bagging predictors. Mach. Learn..

[bib0006] Breiman L. (1996). Stacked regressions. Mach. Learn..

[bib0007] Cameron E., Battle K.E., Bhatt S., Weiss D.J., Bisanzio D., Mappin B., Dalrymple U., Hay S.I., Smith D.L., Griffin J.T. (2015). Defining the relationship between infection prevalence and clinical incidence of *Plasmodium falciparum* malaria. Nat. Commun..

[bib0008] Cibulskis R.E., Aregawi M., Williams R., Otten M., Dye C. (2011). Worldwide incidence of malaria in 2009: estimates, time trends, and a critique of methods. PLoS Med..

[bib0009] Colombian National Institute of Health, 2016. Sistema Nacional de Vigilancia en Salud Pública. online data resource.

[bib0010] Friedman J.H., Stuetzle W. (1981). Projection pursuit regression. J. Am. Stat. Assoc..

[bib0011] Fuglstad G.-A., Simpson D., Lindgren F., Rue H. (2018). Constructing priors that penalize the complexity of Gaussian random fields. J. Am. Stat. Assoc..

[bib0012] Gaughan A.E., Stevens F.R., Linard C., Jia P., Tatem A.J. (2013). High resolution population distribution maps for Southeast Asia in 2010 and 2015. PLoS ONE.

[bib0013] Gething P.W., Van Boeckel T.P., Smith D.L., Guerra C.A., Patil A.P., Snow R.W., Hay S.I. (2011). Modelling the global constraints of temperature on transmission of plasmodium falciparum and P. vivax. Parasites Vectors.

[bib0014] Guerra C.A., Hay S.I., Lucioparedes L.S., Gikandi P.W., Tatem A.J., Noor A.M., Snow R.W. (2007). Assembling a global database of malaria parasite prevalence for the malaria atlas project. Malar. J..

[bib0015] Hao T., Elith J., Guillera-Arroita G., Lahoz-Monfort J.J. (2019). A review of evidence about use and performance of species distribution modelling ensembles like biomod. Divers. Distrib..

[bib0016] Indonesia Ministry of Health, 2013. State health reports.

[bib0017] Johnson O., Diggle P., Giorgi E. (2019). A spatially discrete approximation to log-Gaussian Cox processes for modelling aggregated disease count data. Stat. Med..

[bib0018] Kristensen K., Nielsen A., Berg C.W., Skaug H., Bell B.M. (2016). TMB: Automatic differentiation and Laplace approximation. J. Stat. Softw..

[bib0019] Kuhn, M., Wing, J., Weston, S., Williams, A., Keefer, C., Engelhardt, A., Cooper, T., Mayer, Z., Kenkel, B., the R Core Team, Benesty, M., Lescarbeau, R., Ziem, A., Scrucca, L., Tang, Y., Candan, C., Hunt., T., 2017. caret: Classification and regression training. R package version 6.0-76.

[bib0020] Law H.C., Sejdinovic D., Cameron E., Lucas T.C., Flaxman S., Battle K., Fukumizu K. (2018). Advances in Neural Information Processing Systems.

[bib0021] Li Y., Brown P., Gesink D.C., Rue H. (2012). Log Gaussian Cox processes and spatially aggregated disease incidence data. Stat. Methods Med. Res..

[bib0022] Linard C., Gilbert M., Snow R.W., Noor A.M., Tatem A.J. (2012). Population distribution, settlement patterns and accessibility across Africa in 2010. PLoS ONE.

[bib0023] Lindgren F., Rue H. (2015). Bayesian spatial modelling with R-INLA. J. Stat. Softw..

[bib0024] NASA, 2018. Gridded population of the world (GPW), v4.

[bib0025] Pfeffer D.A., Lucas T.C.D., May D., Harris J., Rozier J., Twohig K.A., Dalrymple U., Guerra C.A., Moyes C.L., Thorn M. (2018). malariaAtlas: an R interface to global malariometric data hosted by the Malaria Atlas Project. Malar. J..

[bib0026] Pigott D.M., Golding N., Mylne A., Huang Z., Henry A.J., Weiss D.J., Brady O.J., Kraemer M.U., Smith D.L., Moyes C.L. (2014). Mapping the zoonotic niche of Ebola virus disease in Africa. Elife.

[bib0027] R Core Team, 2018. R: A language and environment for statistical computing. R Foundation for Statistical Computing. Vienna, Austria.

[bib0028] Rakotorahalahy, A. J., 2009. NMCP Madagascar monthly case data 2013–2014. Pers. Comms. Programme National de Lutte contre le Paludisme.

[bib0029] Richardson S., Gilks W.R. (1993). Conditional independence models for epidemiological studies with covariate measurement error. Stat. Med..

[bib0030] Ridgeway et al., 2017. gbm: Generalized boosted regression models. R package version 2.1.3.

[bib0031] Senegal Ministry of Health, 2009. Senegal Statistical Yearbook 2009.

[bib0032] Shearer F.M., Huang Z., Weiss D.J., Wiebe A., Gibson H.S., Battle K.E., Pigott D.M., Brady O.J., Putaporntip C., Jongwutiwes S. (2016). Estimating geographical variation in the risk of zoonotic *Plasmodium knowlesi* infection in countries eliminating malaria. PLoS Negl. Trop. Dis..

[bib0033] Sill, J., Takács, G., Mackey, L., Lin, D., 2009. Feature-weighted linear stacking. arXiv:0911.0460.

[bib0034] Simpson D., Rue H., Riebler A., Martins T.G., Sørbye S.H. (2017). Penalising model component complexity: a principled, practical approach to constructing priors. Stat. Sci..

[bib0035] Smith D.L., Guerra C.A., Snow R.W., Hay S.I. (2007). Standardizing estimates of the *Plasmodium falciparum* parasite rate. Malar. J..

[bib0036] Sorichetta A., Hornby G.M., Stevens F.R., Gaughan A.E., Linard C., Tatem A.J. (2015). High-resolution gridded population datasets for Latin America and the Caribbean in 2010, 2015, and 2020. Sci. Data.

[bib0037] Sturrock H.J., Bennett A.F., Midekisa A., Gosling R.D., Gething P.W., Greenhouse B. (2016). Mapping malaria risk in low transmission settings: challenges and opportunities. Trends Parasitol..

[bib0038] Sturrock H.J., Cohen J.M., Keil P., Tatem A.J., Le Menach A., Ntshalintshali N.E., Hsiang M.S., Gosling R.D. (2014). Fine-scale malaria risk mapping from routine aggregated case data. Malar. J..

[bib0039] Taylor B.M., Andrade-Pacheco R., Sturrock H.J. (2017). Continuous inference for aggregated point process data. J. R. Stat. Soc. Ser. A (Statistics in Society).

[bib0040] Venables W.N., Ripley B.D. (2002).

[bib0041] Wang C., Puhan M.A., Furrer R., Group S.S. (2018). Generalized spatial fusion model framework for joint analysis of point and areal data. Spat. Stat..

[bib0042] Weiss D.J., Bhatt S., Mappin B., Van Boeckel T.P., Smith D.L., Hay S.I., Gething P.W. (2014). Air temperature suitability for *Plasmodium falciparum* malaria transmission in Africa 2000–2012: a high-resolution spatiotemporal prediction. Malar. J..

[bib0043] Weiss D.J., Lucas T.C.D., Nguyen M., Nandi A.K., Bisanzio D., Battle K.E., Cameron E., Twohig K.A., Pfeffer D.A., Rozier J.A. (2019). Mapping the global prevalence, incidence, and mortality of *Plasmodium falciparum*, 2000–17: a spatial and temporal modelling study. Lancet.

[bib0044] Weiss D.J., Mappin B., Dalrymple U., Bhatt S., Cameron E., Hay S.I., Gething P.W. (2015). Re-examining environmental correlates of *Plasmodium falciparum* malaria endemicity: a data-intensive variable selection approach. Malar. J..

[bib0045] Weiss D.J., Nelson A., Gibson H.S., Temperley W., Peedell S., Lieber A., Hancher M., Poyart E., Belchior S., Fullman N. (2018). A global map of travel time to cities to assess inequalities in accessibility in 2015. Nature.

[bib0046] Wilson K., Wakefield J. (2018). Pointless spatial modeling. Biostatistics.

[bib0047] Wolpert D.H. (1992). Stacked generalization. Neural Netw..

[bib0048] World Health Organization (2016).

[bib0049] Wright M.N., Ziegler A. (2015). Ranger: a fast implementation of random forests for high dimensional data in C++ and R. arXiv preprint arXiv:1508.04409.

[bib0050] Zou, H., Hastie, T., 2012. elasticnet: Elastic-Net for sparse estimation and sparse PCA. R package version 1.1.

