# Supplementary material for: Improving disaggregation models of malaria incidence by ensembling non-linear models of prevalence
Source: Spat Spatiotemporal Epidemiol. 2022 Jun;41:None. doi: 10.1016/j.sste.2020.100357 (PMC9205339; doi:10.1016/j.sste.2020.100357)
Supplement: Supplementary Data S1 — Supplementary Raw Research Data. This is open data under the CC BY license http://creativecommons.org/licenses/by/4.0/ [file mmc1.pdf]

Supporting information: Improving disaggregation models of malaria incidence by ensembling non-linear models of prevalence

Contents

1 Supplementary methods **2**

1.1 Extended methods for machine learning models 2

2 Global dataset Machine Learning **5**

2.1 Out-of-sample scatter plots 6

2.2 Hyperparameter optimisation 9

3 Colombia (South America) prevalence dataset Machine Learning **11**

3.1 Predictions 11

3.2 Out-of-sample scatter plots 13

3.3 Hyperparameter optimisation 16

4 Indonesia dataset Machine Learning **18**

4.1 Predictions 18

4.2 Out-of-sample scatter plots 20

4.3 Hyperparameter optimisation 23

5 Madagascar dataset Machine Learning **24**

5.1 Predictions 24

5.2 Out-of-sample scatter plots 26

5.3 Hyperparameter optimisation 29

6 Senegal dataset Machine Learning **30**

6.1 Predictions 30

6.2 Out-of-sample scatter plots 32

6.3 Hyperparameter optimisation 35

7 Colinearity between covariates **36**

1. Supplementary methods

1.1. Extended methods for machine learning models

We trained five machine learning models to each prevalence dataset. These models were elastic net, projection pursuit regression, neural networks, random forest and boosted regression trees. All models were trained on untransformed prevalence data with any predictions below zero being set at zero. For tree based models, transforming the data makes almost no difference. For the other models, training on empirical logit transformed data was considered but as the final performance metric (correlation between predicted and observed polygon incidence data) was on an absolute scale it was deemed better to train the models on absolute scales. For all models, the sample size of each survey was used as a weight.

Elastic net models are penalised, linear regression models. They find the regression parameters that optimise

$$\hat{\beta} = \underset{\beta}{\operatorname{argmin}} \sum_{i=1}^{N} w_i(y_i - X_i\beta)^2 + \lambda \left((1-\alpha)\beta^2 + \alpha|\beta|\right)$$

where β is a vector of regression parameters, y_i is the prevalence response data, X_i is a vector of environmental covariates and w_i is the weight given by the sample size. λ is the penalty term and α controls the relative contribution of the l1 and l2 penalties. With $\alpha = 0$ the model collapses to ridge regression while with $\alpha = 1$ the model is equivalent to the LASSO. α and λ are selected by a cross validation with candidate values given by a grid search. We examined 15 values for α taken evenly between 0.05 and 1. We examined 15 values for λ taken as 14 values spread evenly on a log10 scale between -4 and -1 and additionally considering $\lambda = 0$ which gives the least squares estimate.

Projection pursuit regression fits a number of smooth functions to linear combinations of all the covariates. It involves finding regression parameters β in the model

$$y_i = \beta_0 + \sum_{j=1}^{r} f_j(\beta_j X_i)$$

where the f_j's are smoothing functions, X_i is the vector of covariates and r is the number of smooth terms to be fitted (a hyperparameter selected by cross-validation). The model is fitted iteratively r times with the model being fitted to the residuals of the previous models at each iteration. We considered rß in the range 1 to 15.

We fitted neural networks with a single hidden layer, logistic activation functions for the hidden units and a linear activation function for the output. This architecture, with h hidden units and c covariates, can be written as

$$y_i = \beta_0 + \sum_h \beta_{ho} \operatorname{logit}^{-1}\left(\beta_{0h} + \sum_c \beta_{ih} X_{ic}\right)$$

with β_0 being the intercept, β_{0h} being the intercept for each hidden unit, β_{ih} being the weights between the inputs and hidden units and β_{ho} being the weights between the hidden units and the outputs. The nnet package optimises this model using back-propogation and includes an additional hyperparameter, 'decay', that is a penalty on the sum of the squares of the weights. We considered a grid of hyperparameters. For the number of hidden units we considered 15 equally spaced values between 1 and 29. We examined 15 values for *decay* taken as 14 values spread evenly on a log10 scale between -4 and -1 and additionally considering $\lambda = 0$.

The Random Forest algorithm creates an ensemble of regression trees. To control overfitting each tree is fitted to a bootstrap sample of the data. To further control overfitting, at each node of each tree, mtry random selected covariates are considered instead of the full number of covariates. For each node in a tree the covariate and split point is found which minimises the sum of the variances in the nodes. However, we consider two further variants of this rule. We consider "extremely randomised trees" in which the split point for each covariate is drawn uniformly randomly. We then consider "conditional inference trees" in which after a split point is chosen, a significance test is performed and the split point is only used if the data on each side of the split are significantly different (with a significance threshold of 0.5). The trees are gown until all nodes reach a minimum node size. The split rule and the parameters mtry and the minimal node size are chosen by cross-validation. We considered a random search of 15 parameter sets.

Finally, boosted regression trees also makes an ensemble of trees. The trees are built sequentially and for each successive tree, the data are weighted by the size of the residual between the observed data and the prediction from all the previous trees. The XGBoost implementation of boosted

regression trees contains a number of hyperparameters: nrounds is the number of additive trees, max depth is the maximum depth of each tree, eta reduces the additional weight given to poorly predicted data points, gamma sets the minimum loss reduction needed for a split to be allowed, for each tree, a proportion colsamplebytree of covariates are considered, min child weight causes the growing of the tree to stop when a node has this many data points and finally subsample is a proportion of the data to randomly sample to use in training of each tree. Again we used a random search and considered 15 parameter sets.

2. Global dataset Machine Learning

2.1. Out-of-sample scatter plots

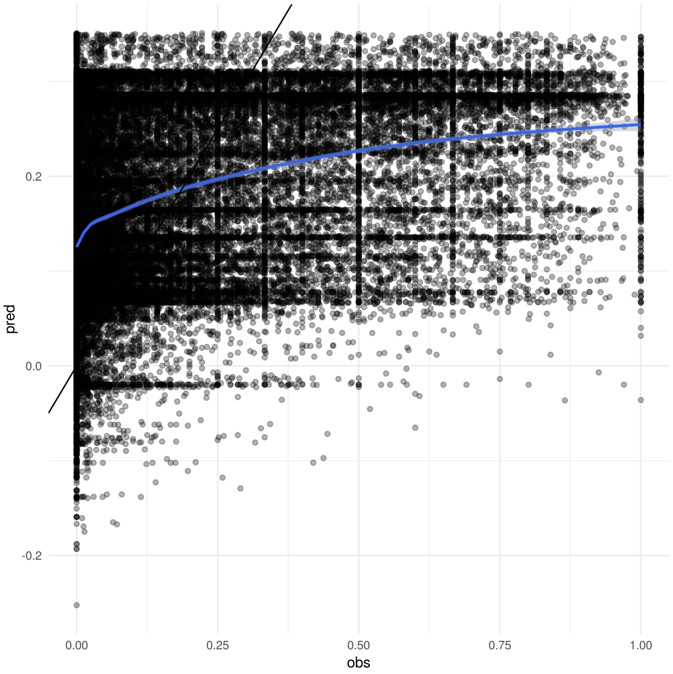

Figure S1: Scatter plot of predictions and held out observed data for the neural network trained on the global dataset.

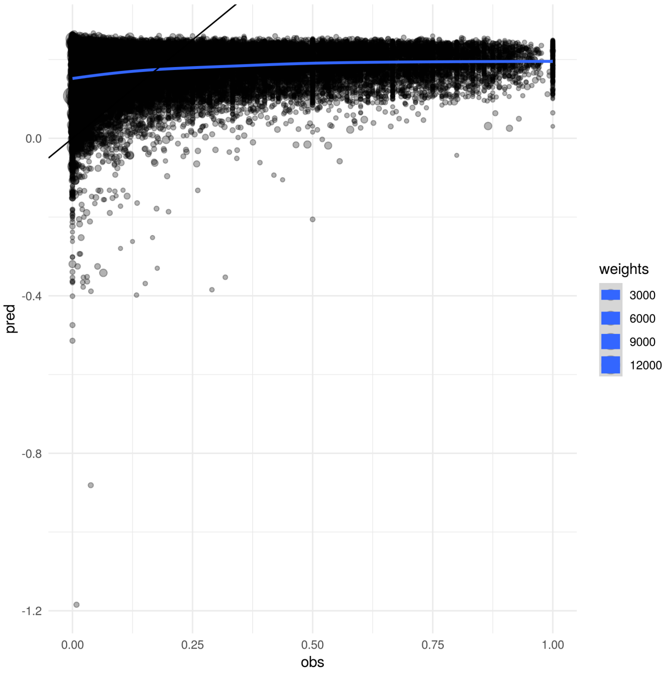

Figure S2: Scatter plot of predictions and held out observed data for the elastic net trained on the global dataset.

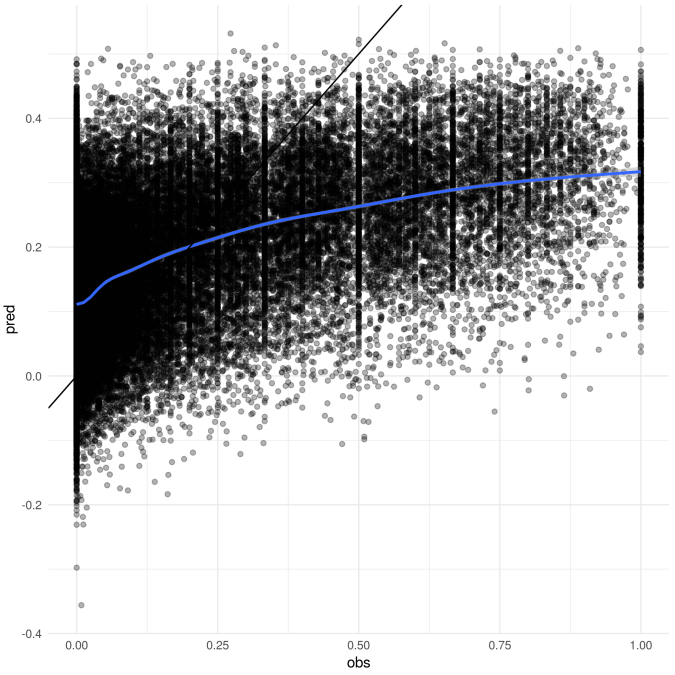

Figure S3: Scatter plot of predictions and held out observed data for the PPR trained on the global dataset.

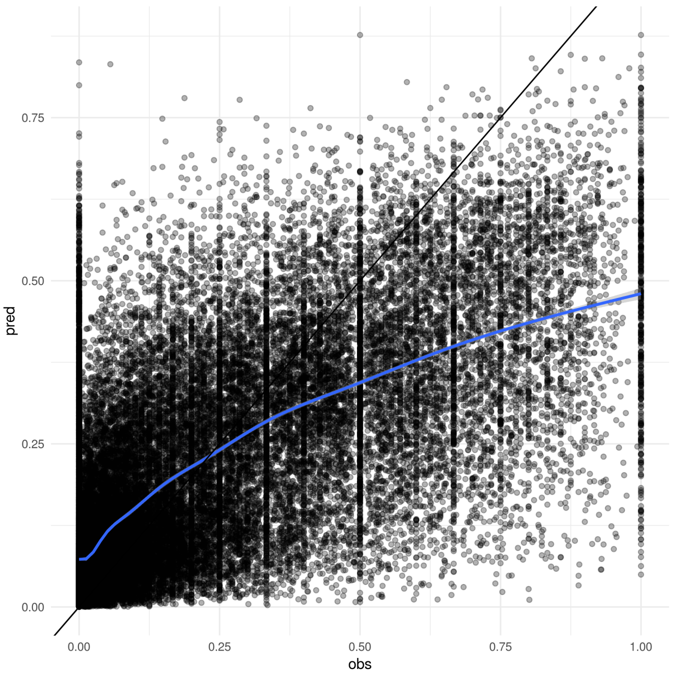

Figure S4: Scatter plot of predictions and held out observed data for the Random Forest trained on the global dataset.

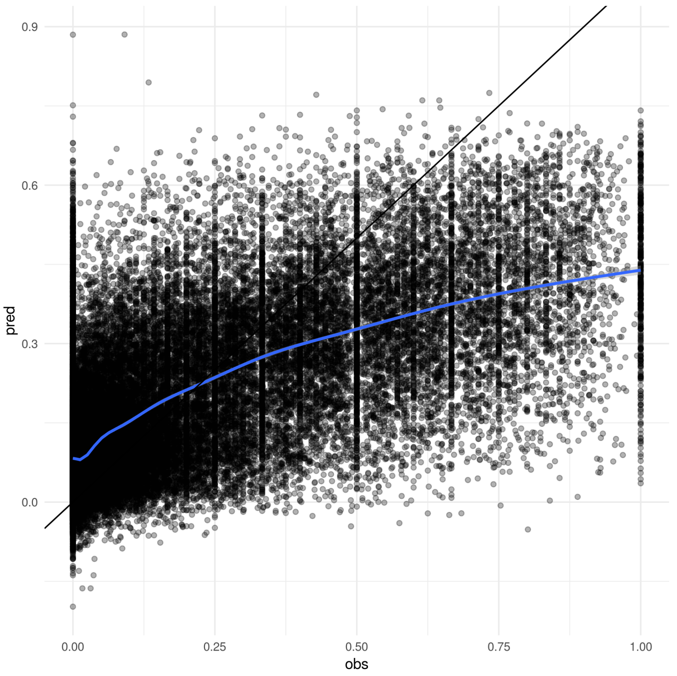

Figure S5: Scatter plot of predictions and held out observed data for the GBM trained on the global dataset.

2.2. Hyperparameter optimisation

As ranger and GBM were tuned with random hyperparameter search, the plots become difficult and are not included.

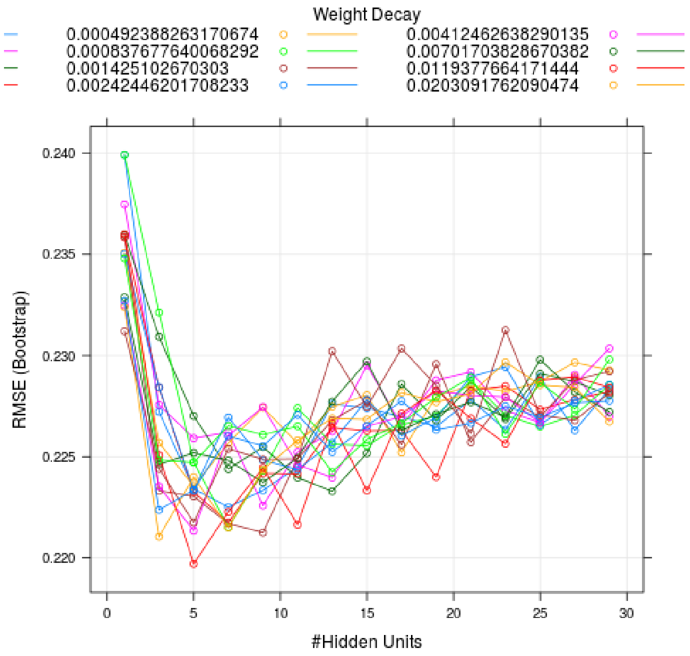

Figure S6: Optimisation for neural network hyperparameters trained on the global dataset.

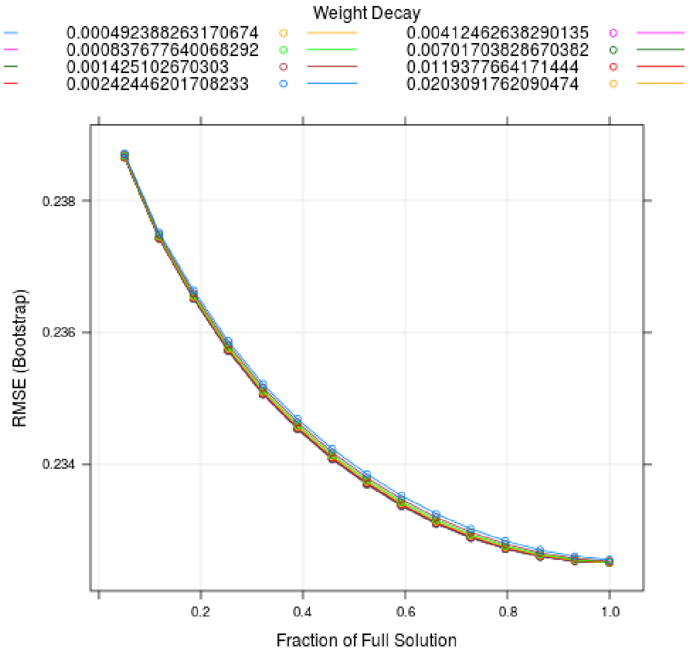

Figure S7: Optimisation for elastic net hyperparameters trained on the global dataset.

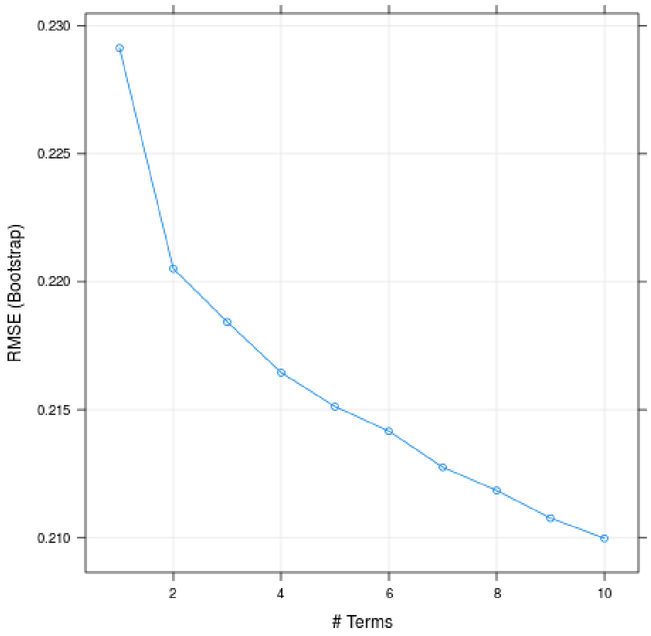

Figure S8: Optimisation for PPR hyperparameters trained on the global dataset.

3. Colombia (South America) prevalence dataset Machine Learning

3.1. Predictions

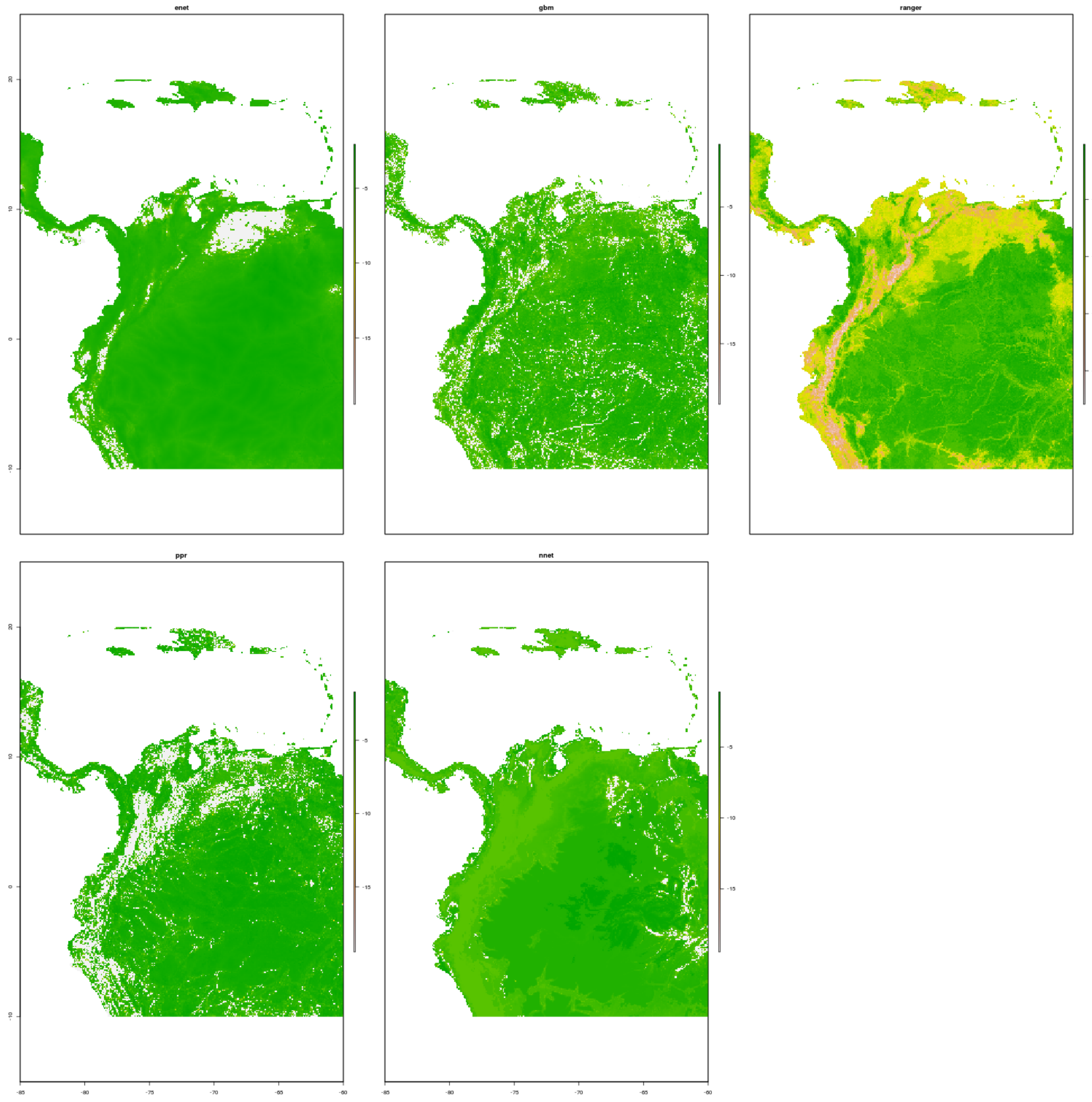

Figure S9: Predictions from machine learning models trained on South American prevalence data.

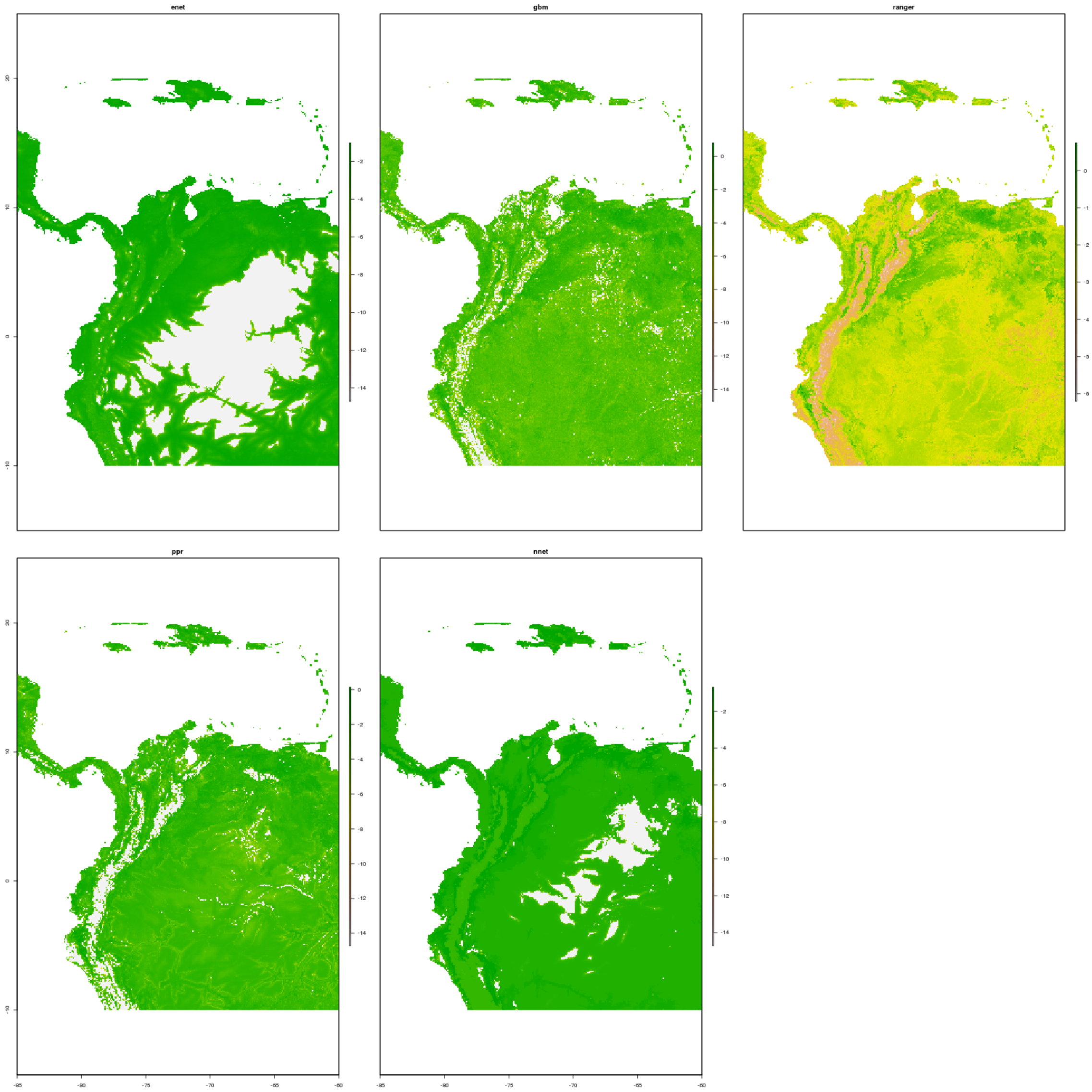

Figure S10: Predictions over Colombia from machine learning models trained on global prevalence data.

3.2. Out-of-sample scatter plots

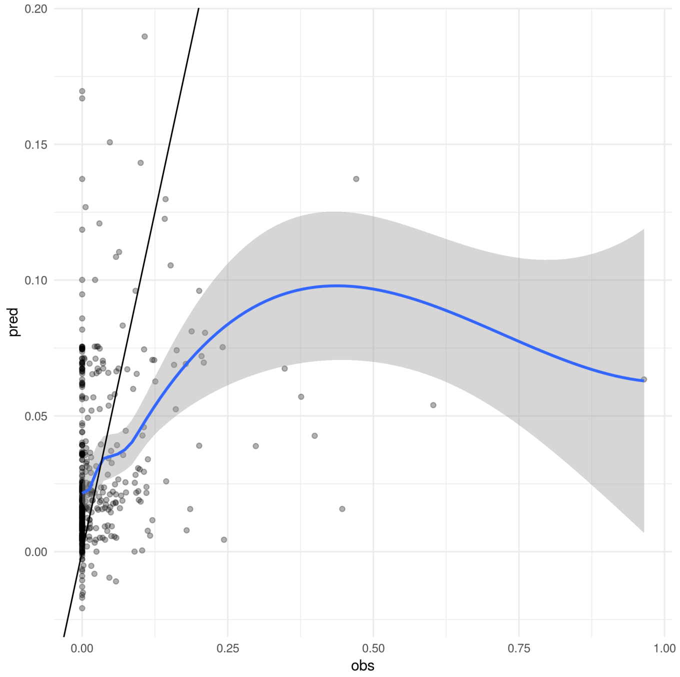

Figure S11: Scatter plot of predictions and held out observed data for the neural network trained on the South America dataset.

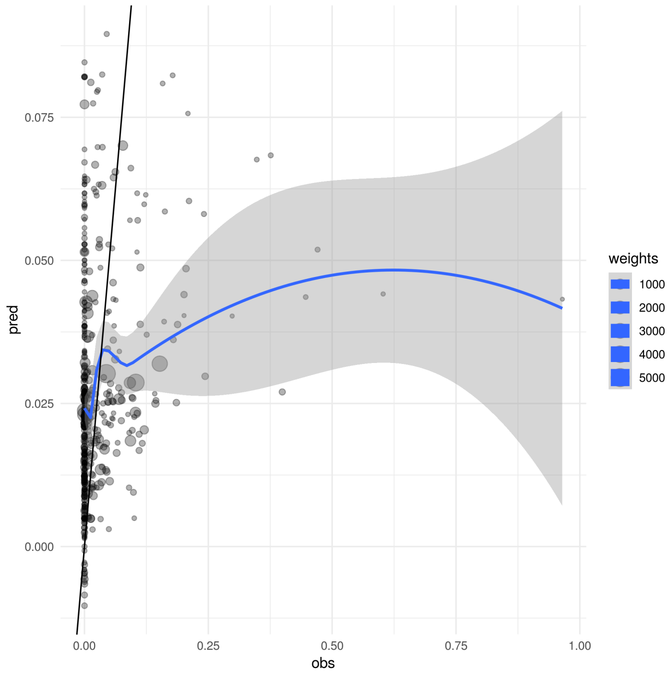

Figure S12: Scatter plot of predictions and held out observed data for the elastic net trained on the South America dataset.

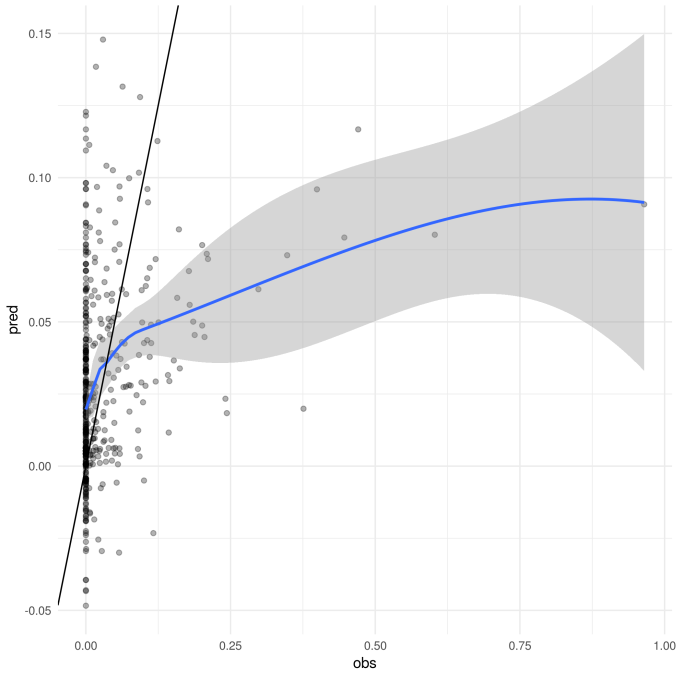

Figure S13: Scatter plot of predictions and held out observed data for the PPR trained on the South America dataset.

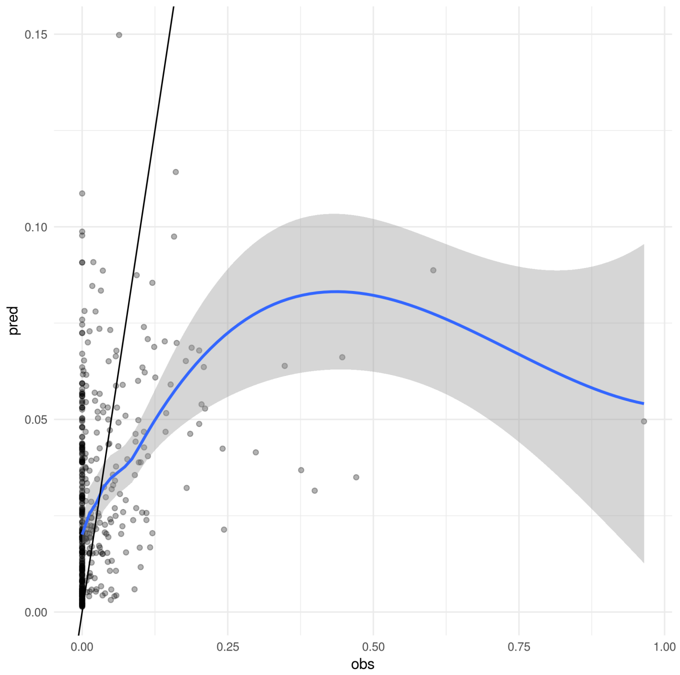

Figure S14: Scatter plot of predictions and held out observed data for the Random Forest trained on the South America dataset.

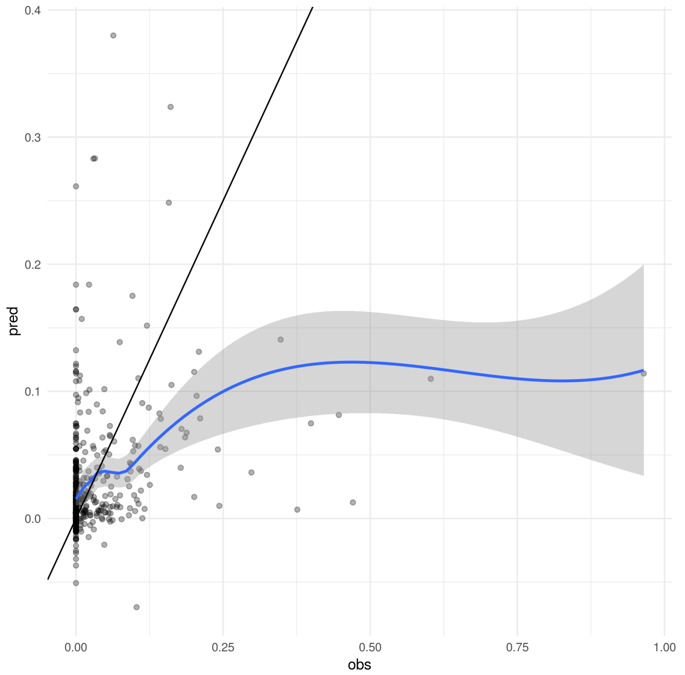

Figure S15: Scatter plot of predictions and held out observed data for the GBM trained on the South America dataset.

3.3. Hyperparameter optimisation

As ranger and GBM were tuned with random hyperparameter search, the plots become difficult and are not included.

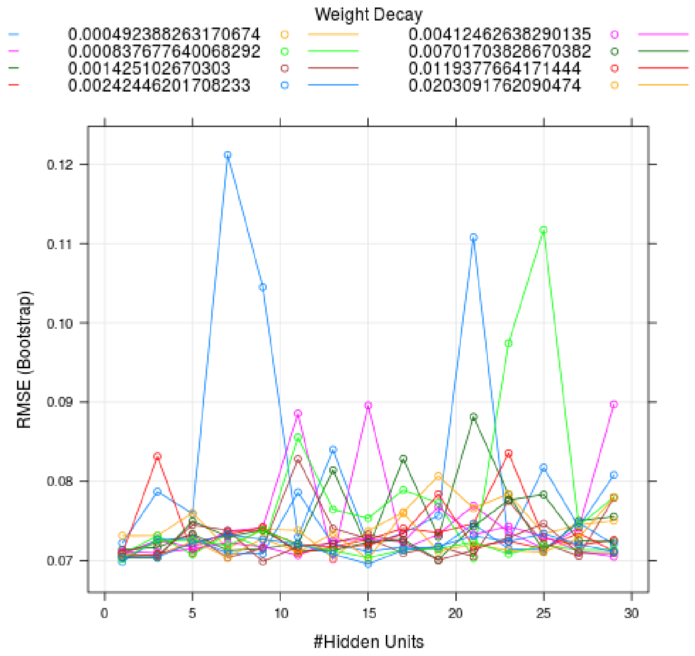

Figure S16: Optimisation for neural network hyperparameters trained on the South America dataset.

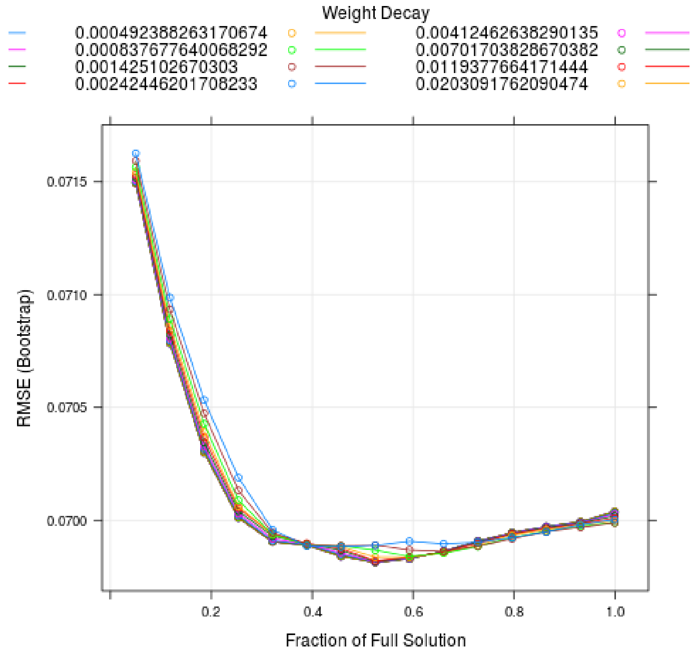

Figure S17: Optimisation for elastic net hyperparameters trained on the South America dataset.

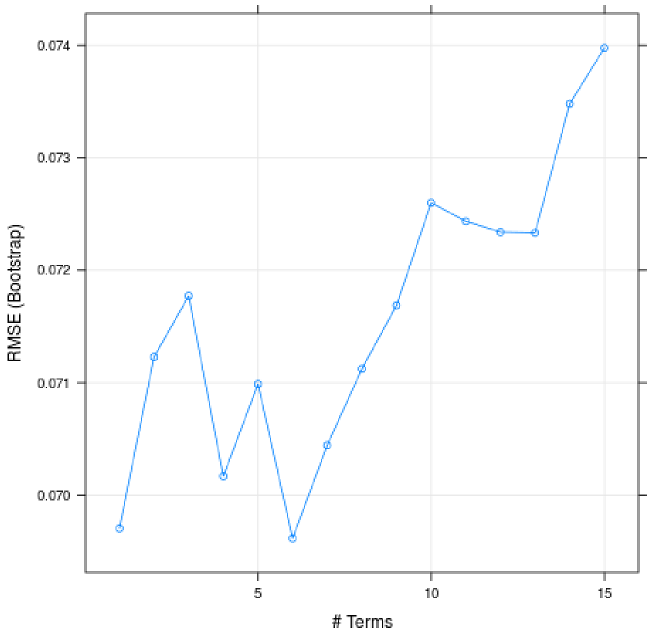

Figure S18: Optimisation for PPR hyperparameters trained on the South America dataset.

4. Indonesia dataset Machine Learning

4.1. Predictions

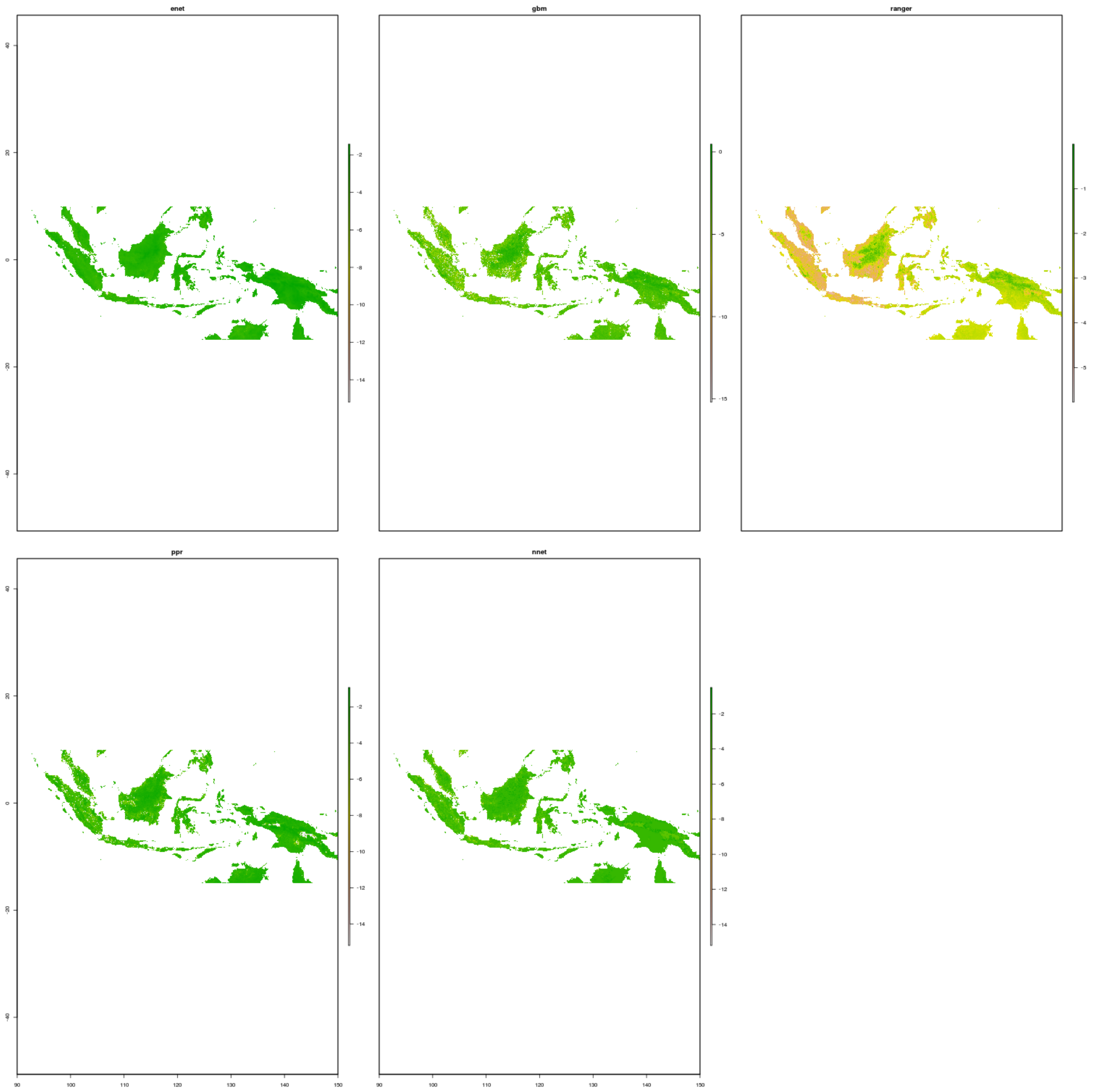

Figure S19: Predictions from machine learning models trained on Indonesian prevalence data.

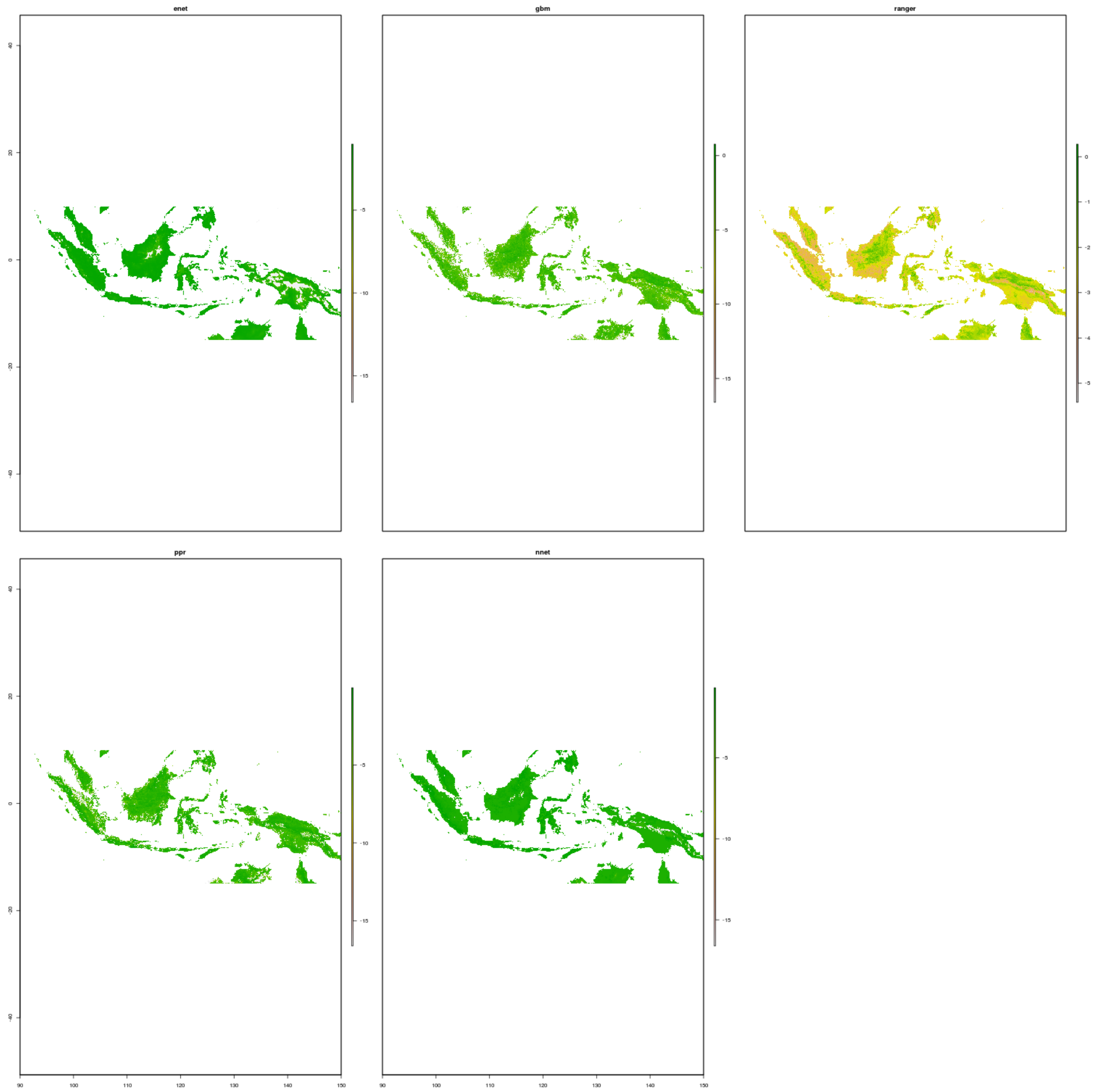

Figure S20: Predictions over Indonesia from machine learning models trained on global prevalence data.

4.2. Out-of-sample scatter plots

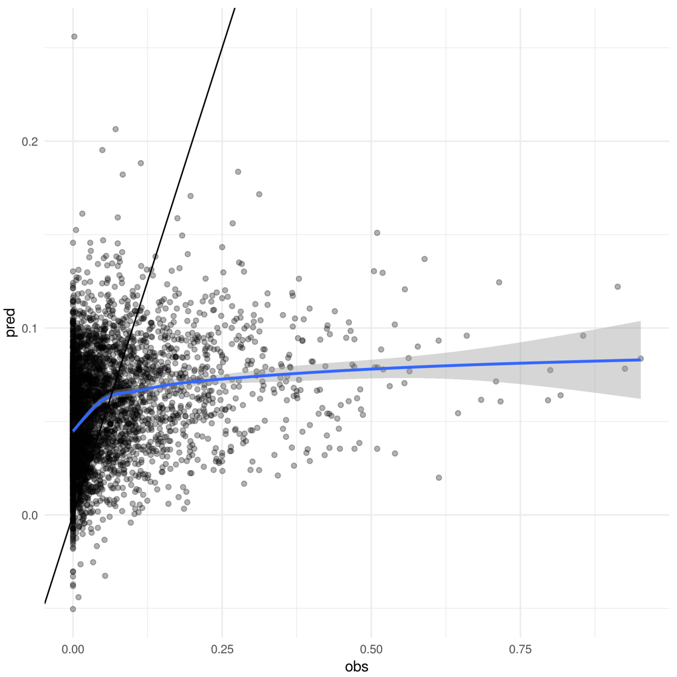

Figure S21: Scatter plot of predictions and held out observed data for the neural network trained on the Indonesia dataset.

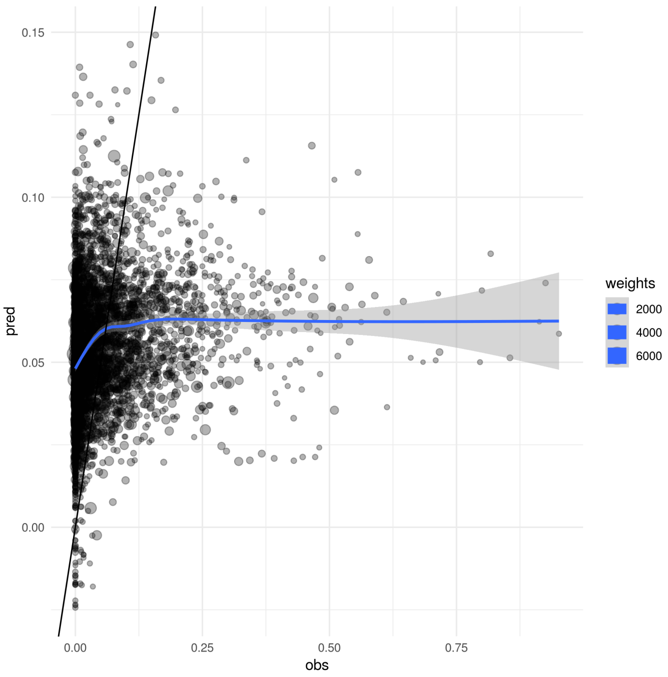

Figure S22: Scatter plot of predictions and held out observed data for the elastic net trained on the Indonesia dataset.

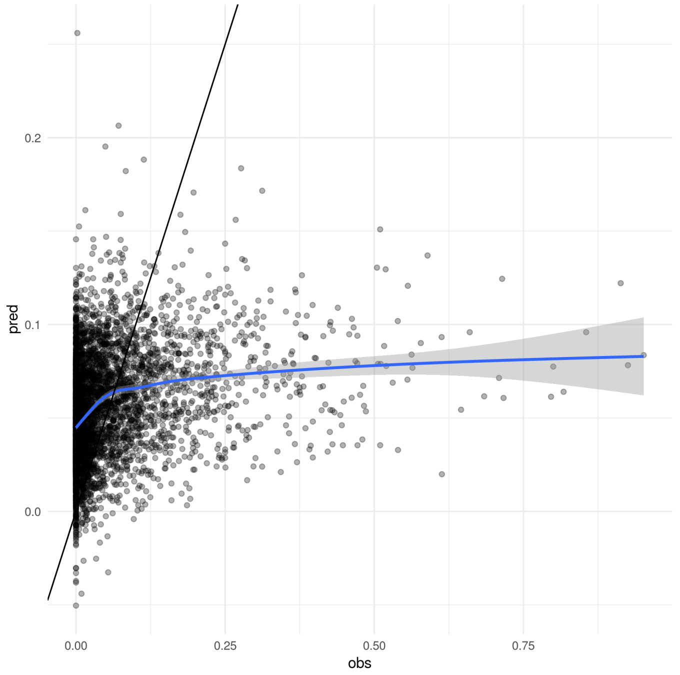

Figure S23: Scatter plot of predictions and held out observed data for the PPR trained on the Indonesia dataset.

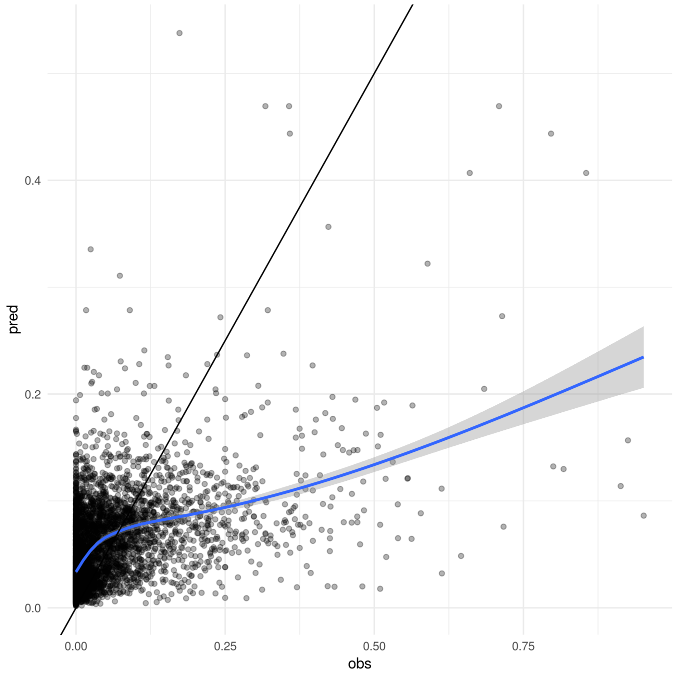

Figure S24: Scatter plot of predictions and held out observed data for the Random Forest trained on the Indonesia dataset.

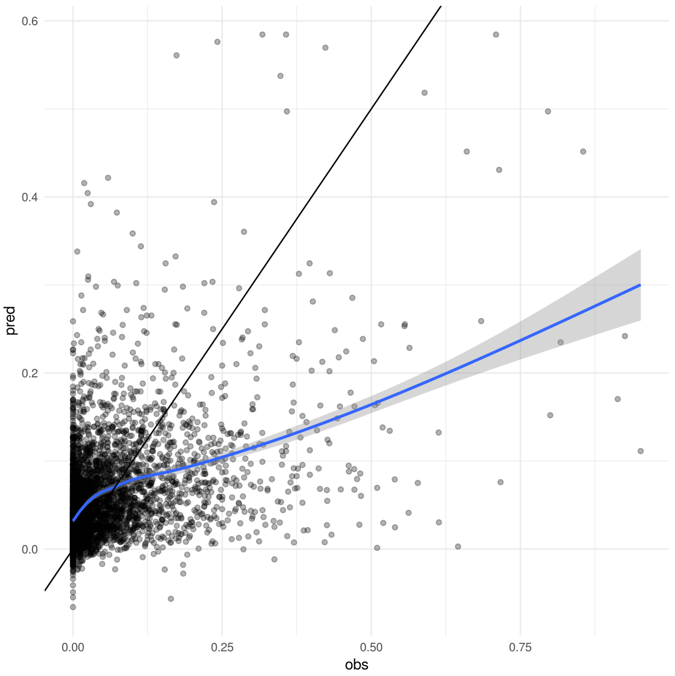

Figure S25: Scatter plot of predictions and held out observed data for the GBM trained on the Indonesia dataset.

4.3. Hyperparameter optimisation

As ranger and GBM were tuned with random hyperparameter search, the plots become difficult and are not included.

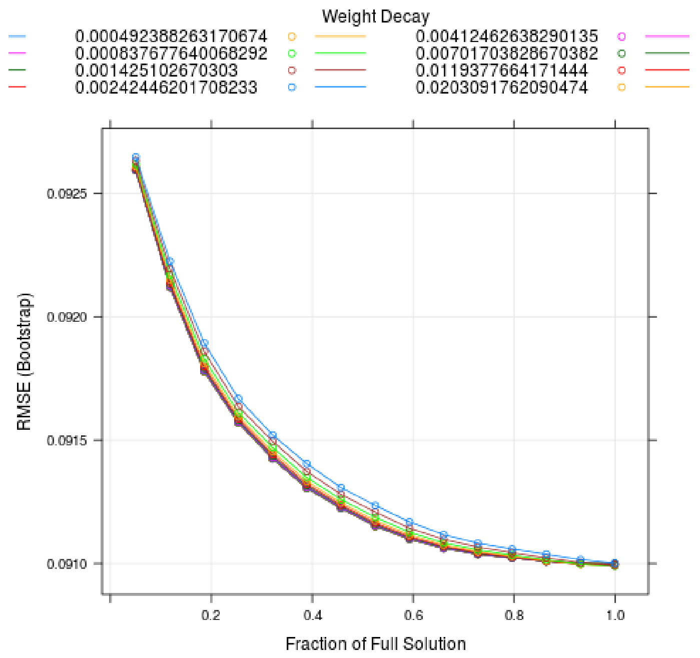

Figure S26: Optimisation for elastic net hyperparameters trained on the Indonesia dataset.

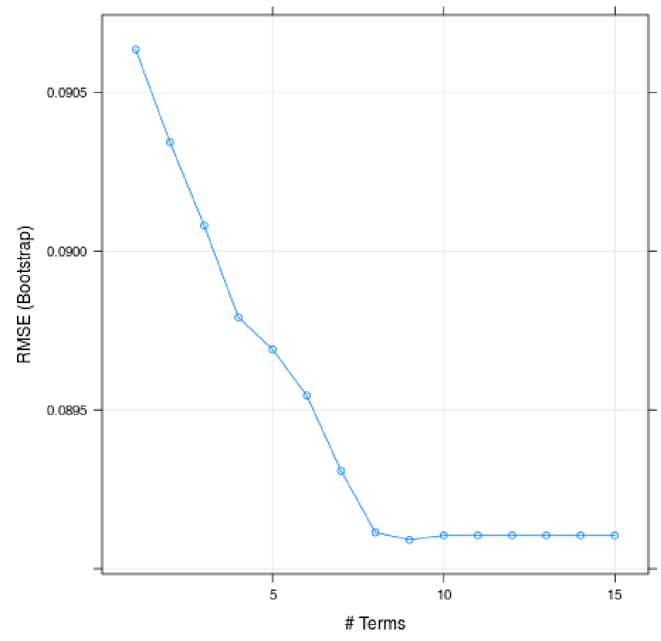

Figure S27: Optimisation for PPR hyperparameters trained on the Indonesia dataset.

5. Madagascar dataset Machine Learning

5.1. Predictions

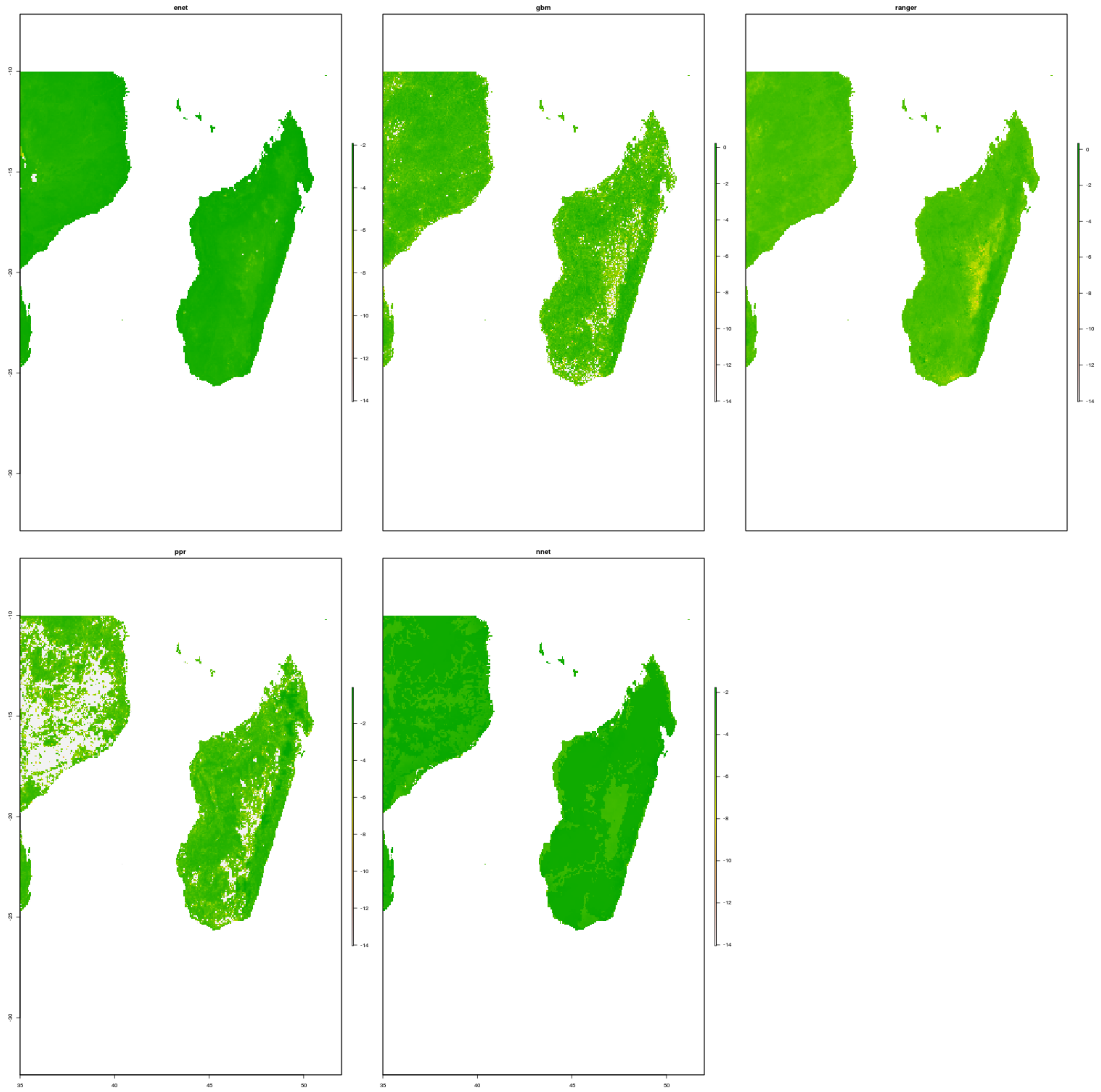

Figure S28: Predictions from machine learning models trained on Malagasy prevalence data.

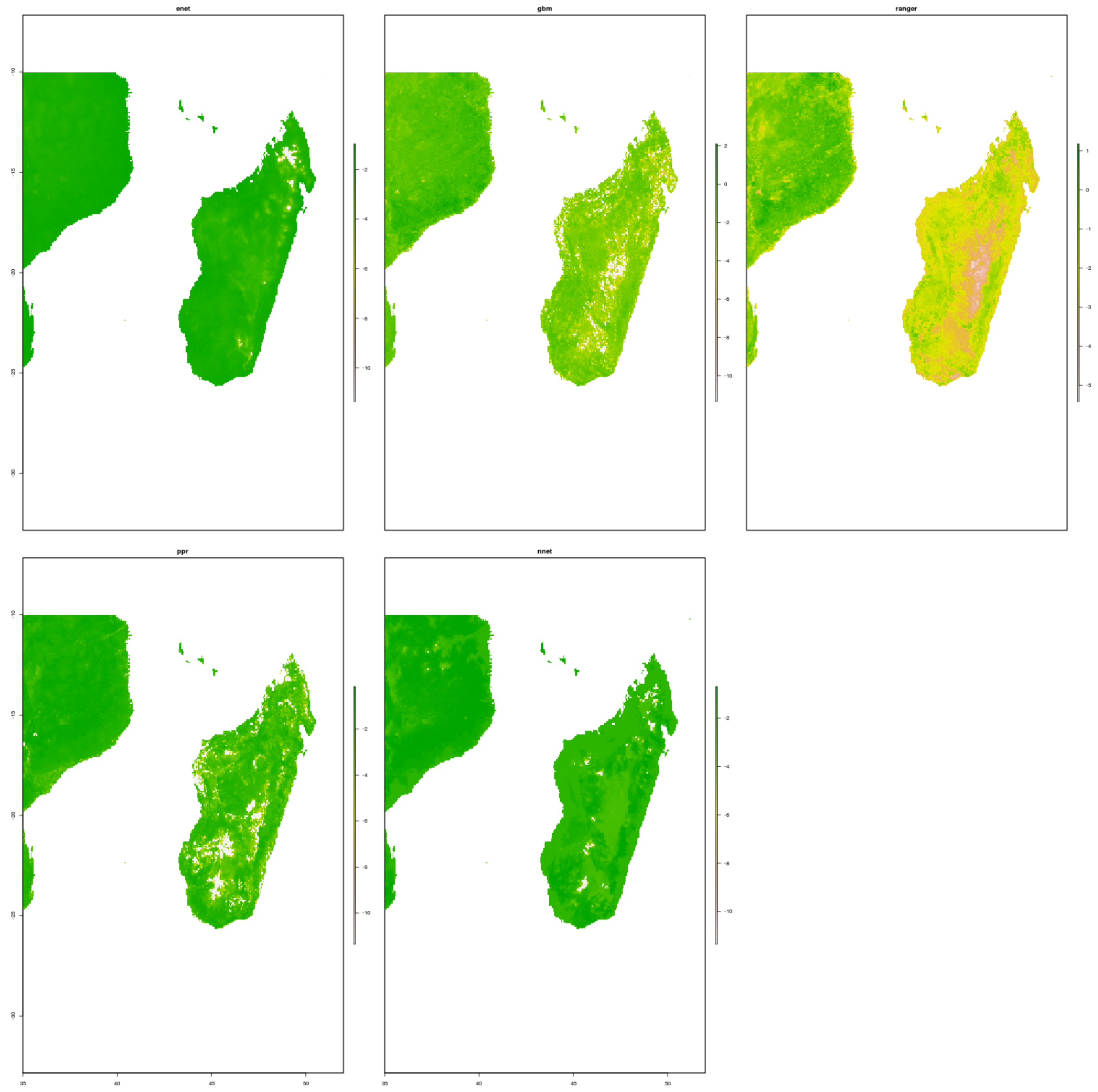

Figure S29: Predictions over Madagascar from machine learning models trained on global prevalence data.

5.2. Out-of-sample scatter plots

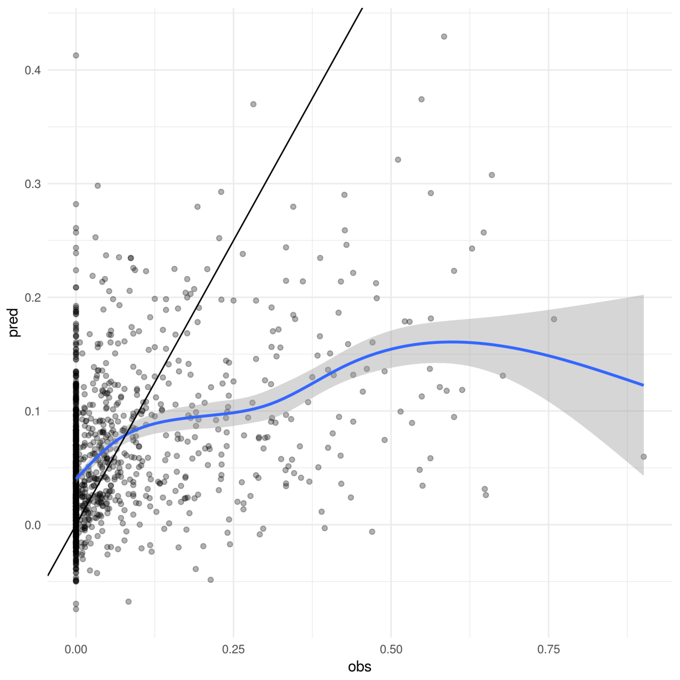

Figure S30: Scatter plot of predictions and held out observed data for the neural network trained on the Madagascar dataset.

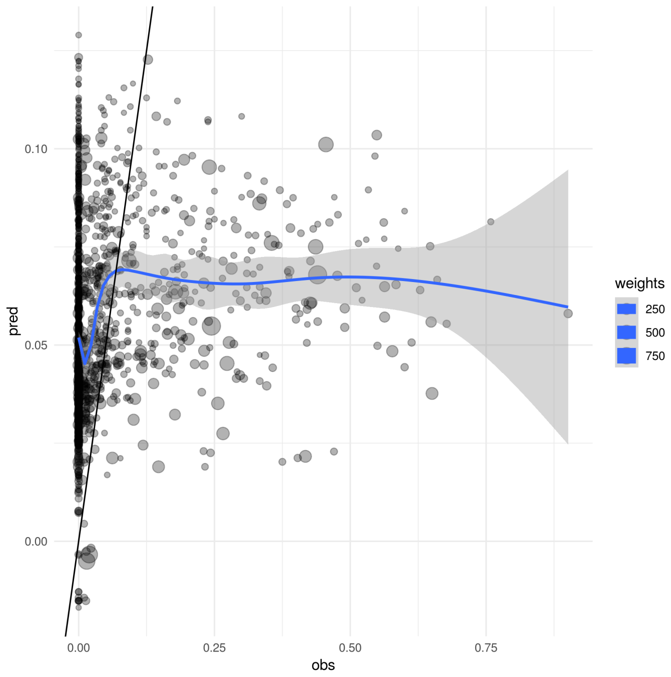

Figure S31: Scatter plot of predictions and held out observed data for the elastic net trained on the Madagascar dataset.

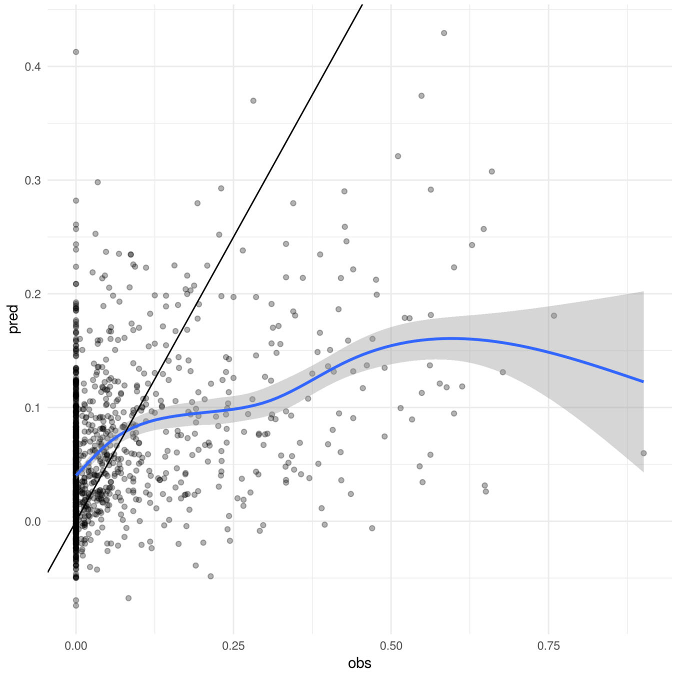

Figure S32: Scatter plot of predictions and held out observed data for the PPR trained on the Madagascar dataset.

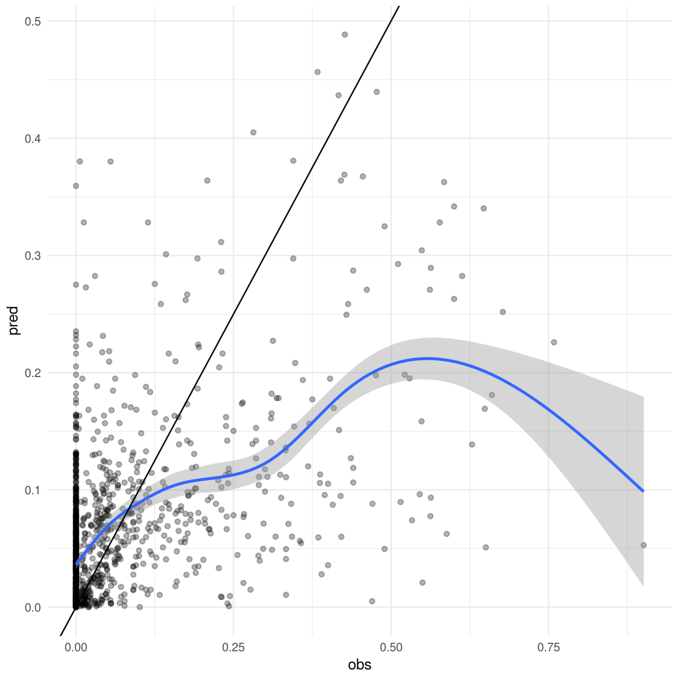

Figure S33: Scatter plot of predictions and held out observed data for the Random Forest trained on the Madagascar dataset.

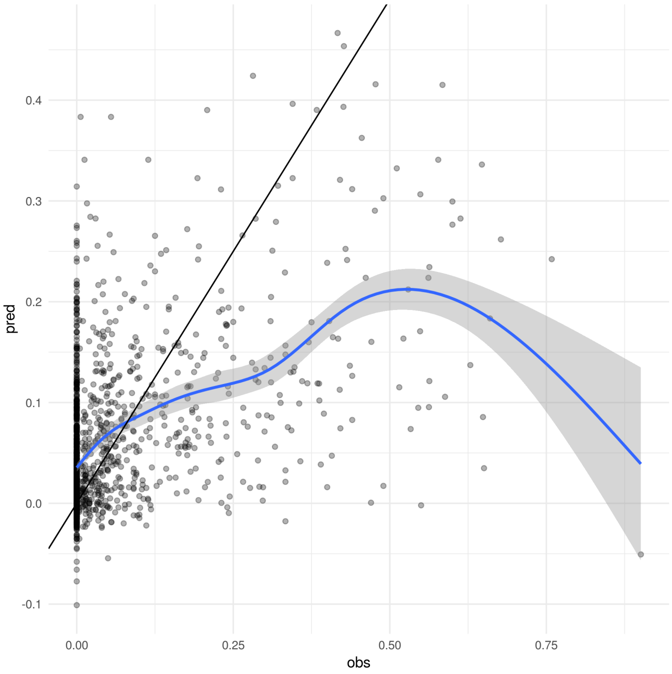

Figure S34: Scatter plot of predictions and held out observed data for the GBM trained on the Madagascar dataset.

5.3. Hyperparameter optimisation

As ranger and GBM were tuned with random hyperparameter search, the plots become difficult and are not included.

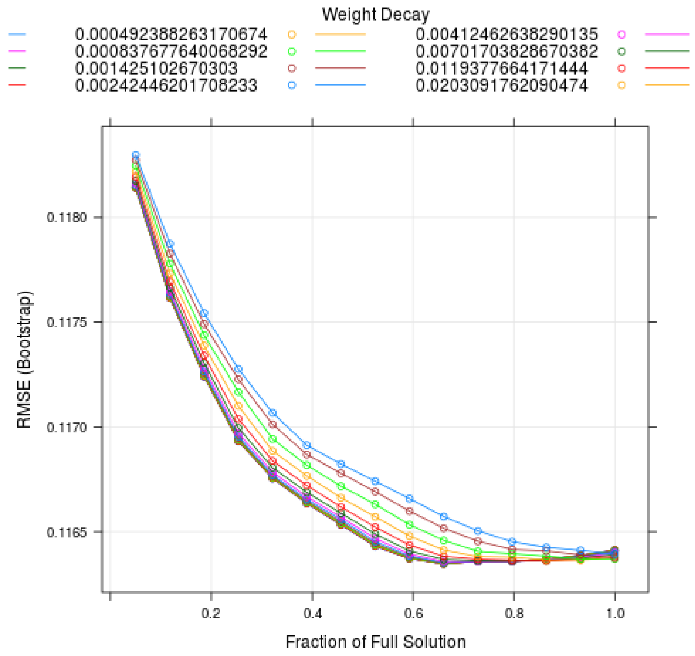

Figure S35: Optimisation for elastic net hyperparameters trained on the Madagascar dataset.

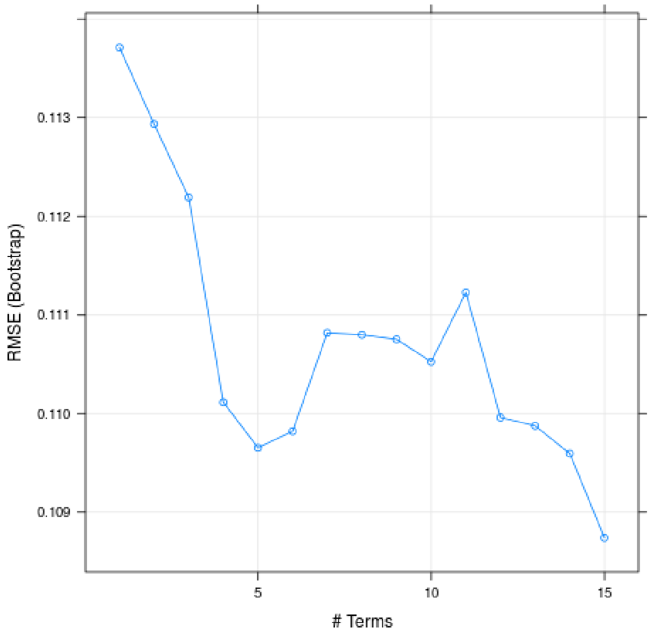

Figure S36: Optimisation for PPR hyperparameters trained on the Madagascar dataset.

6. Senegal dataset Machine Learning

6.1. Predictions

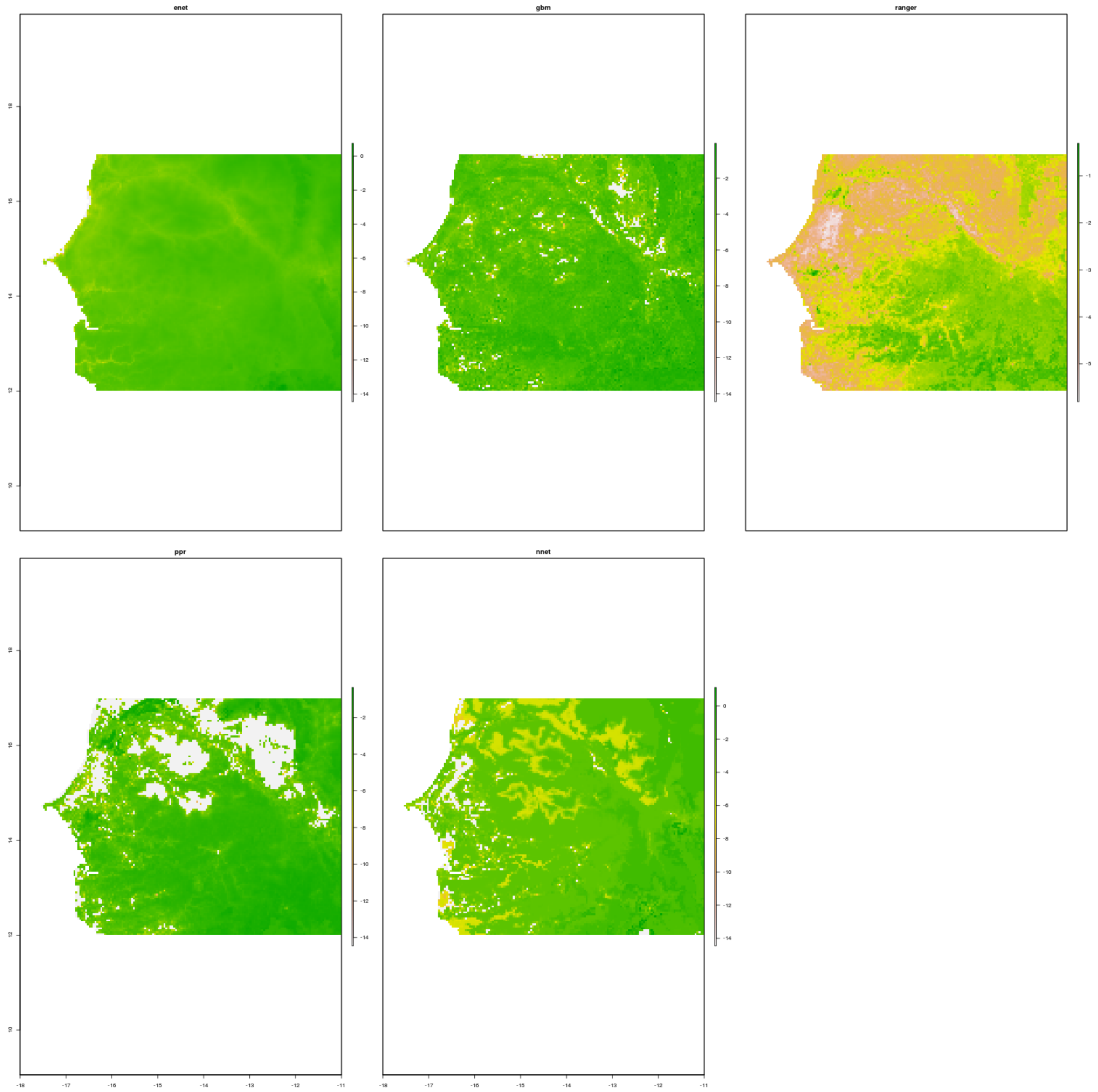

Figure S37: Predictions from machine learning models trained on Senegal prevalence data.

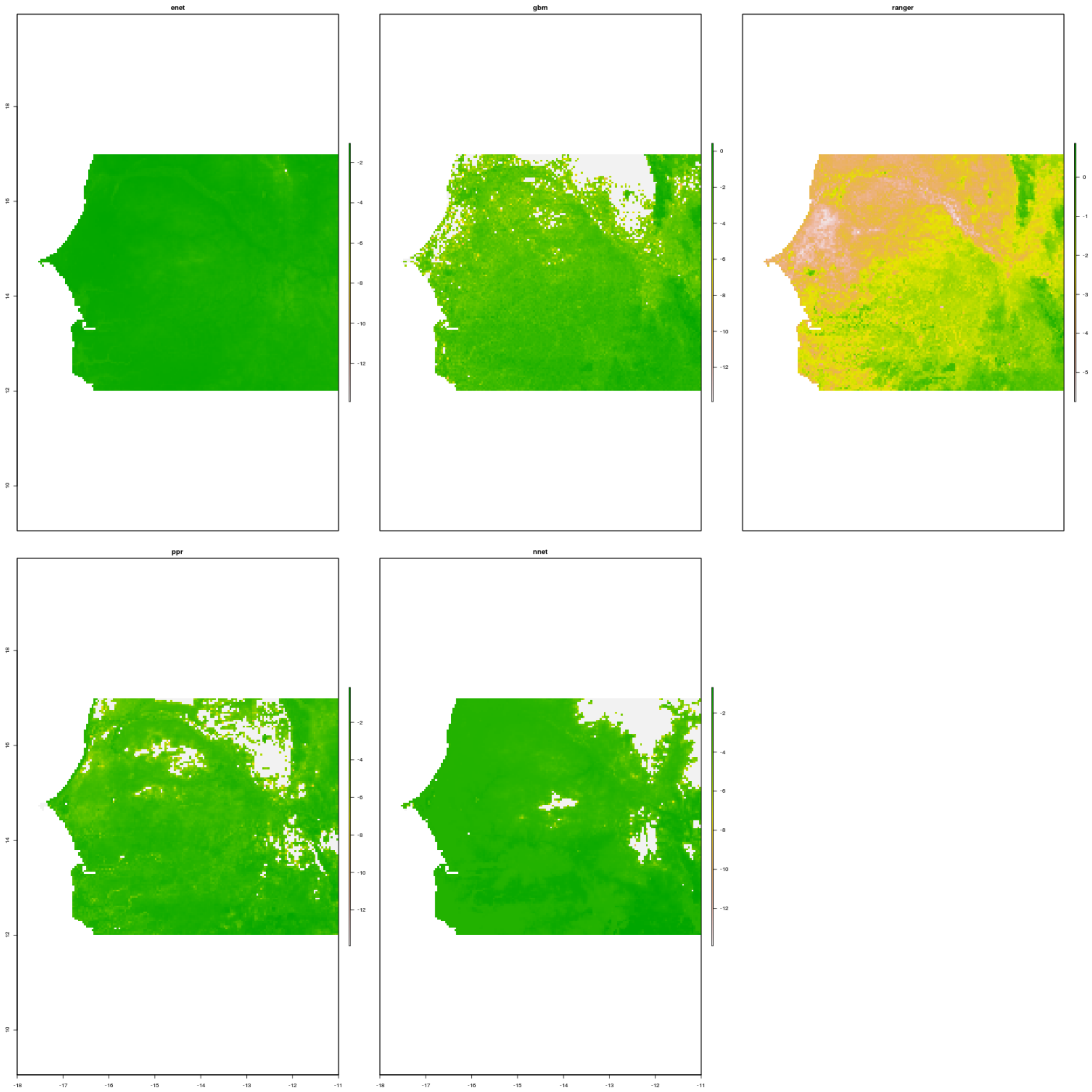

Figure S38: Predictions from machine learning models trained on Senegal prevalence data.

6.2. Out-of-sample scatter plots

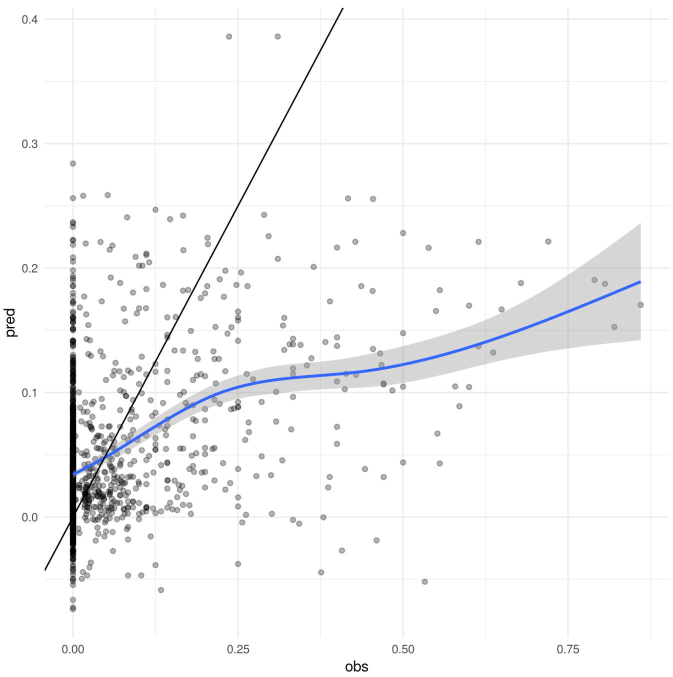

Figure S39: Scatter plot of predictions and held out observed data for the neural network trained on the Senegal dataset.

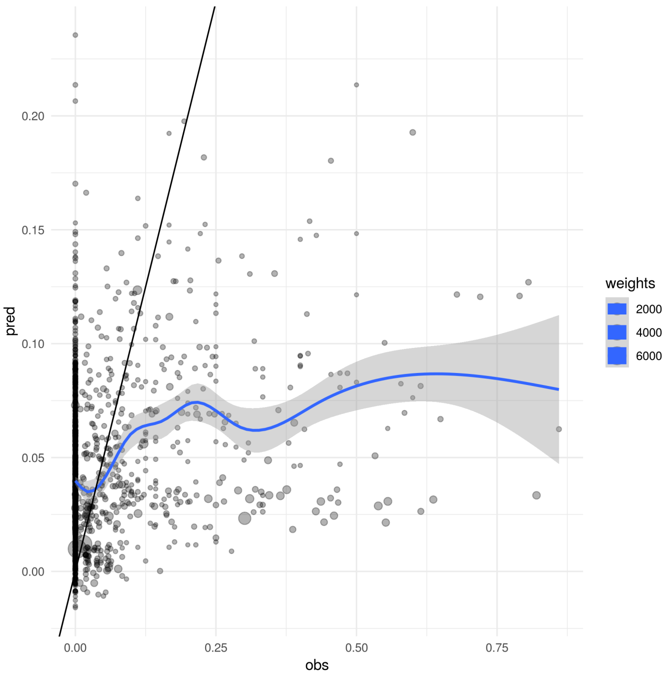

Figure S40: Scatter plot of predictions and held out observed data for the elastic net trained on the Senegal dataset.

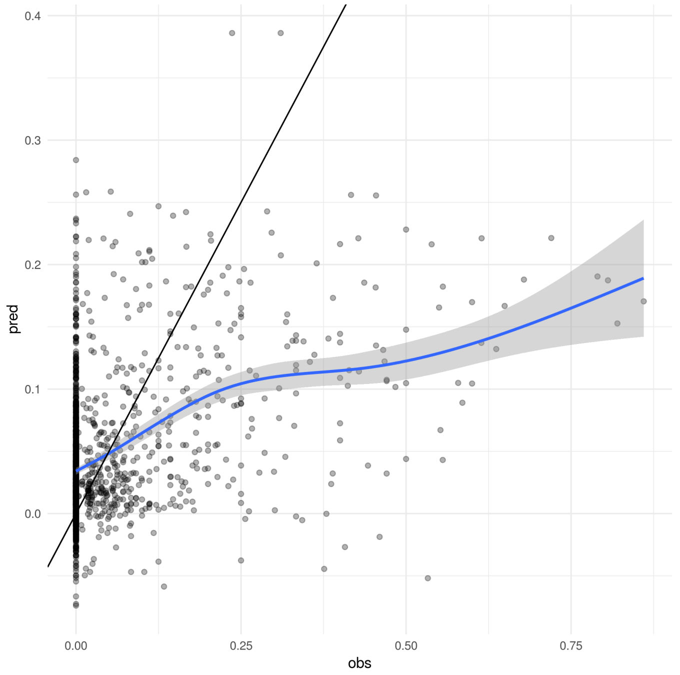

Figure S41: Scatter plot of predictions and held out observed data for the PPR trained on the Senegal dataset.

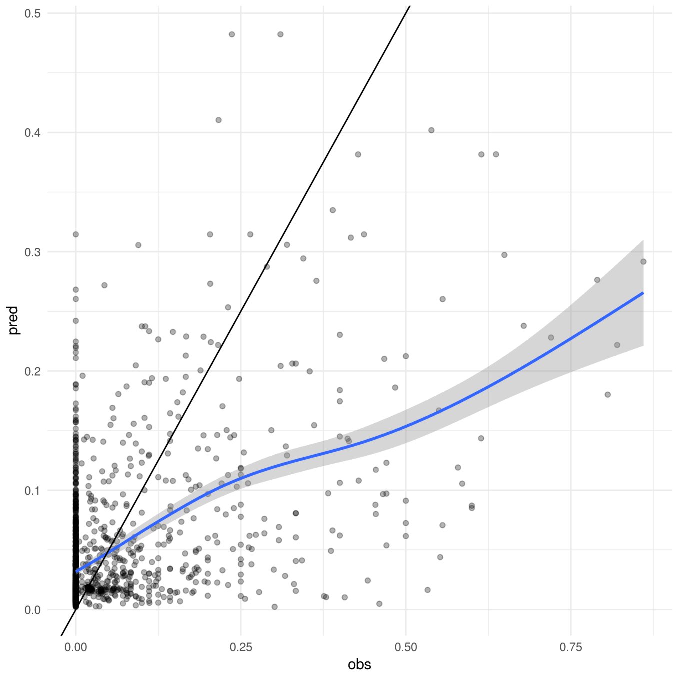

Figure S42: Scatter plot of predictions and held out observed data for the Random Forest trained on the Senegal dataset.

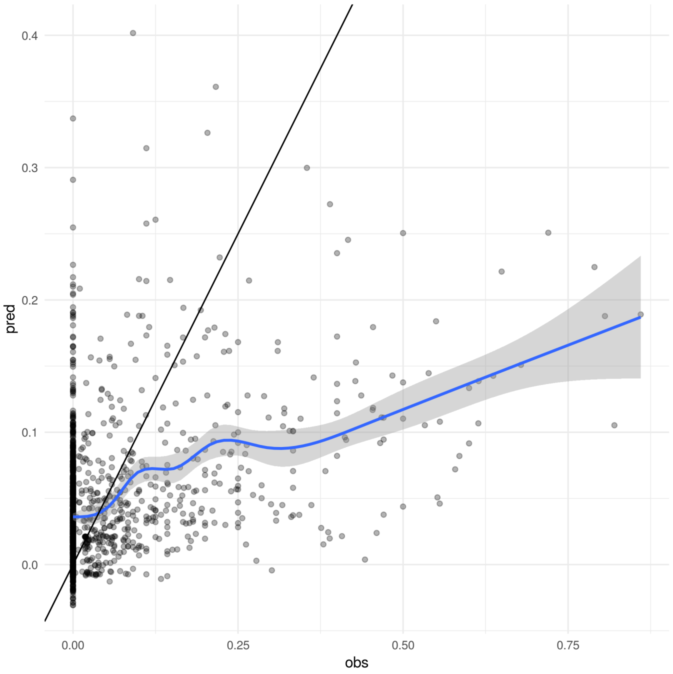

Figure S43: Scatter plot of predictions and held out observed data for the GBM trained on the Senegal dataset.

6.3. Hyperparameter optimisation

As ranger and GBM were tuned with random hyperparameter search, the plots become difficult and are not included.

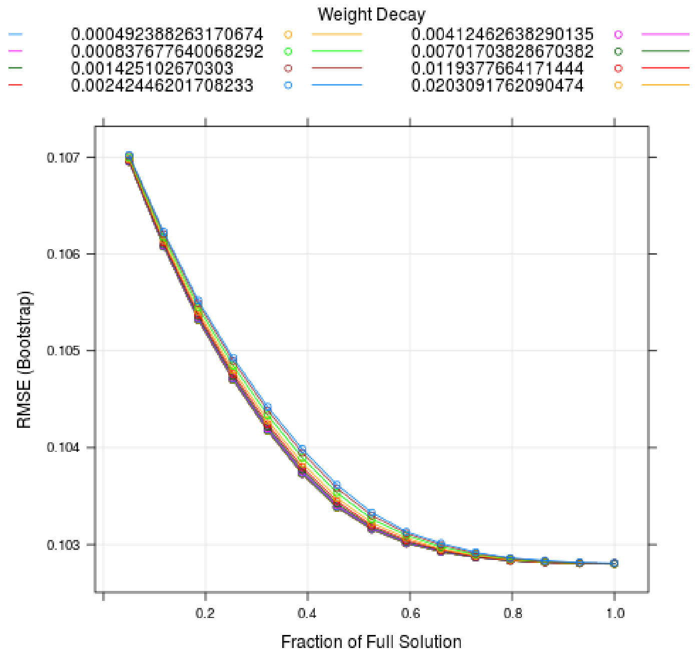

Figure S44: Optimisation for elastic net hyperparameters trained on the Senegal dataset.

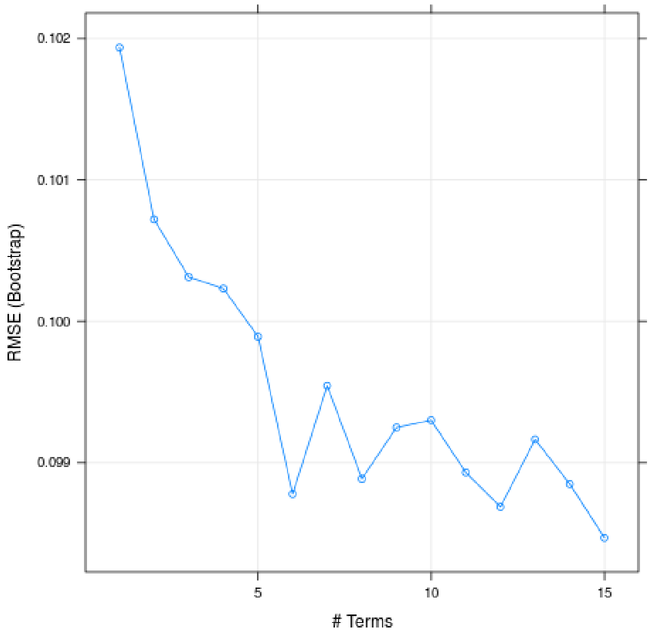

Figure S45: Optimisation for PPR hyperparameters trained on the Senegal dataset.

7. Colinearity between covariates

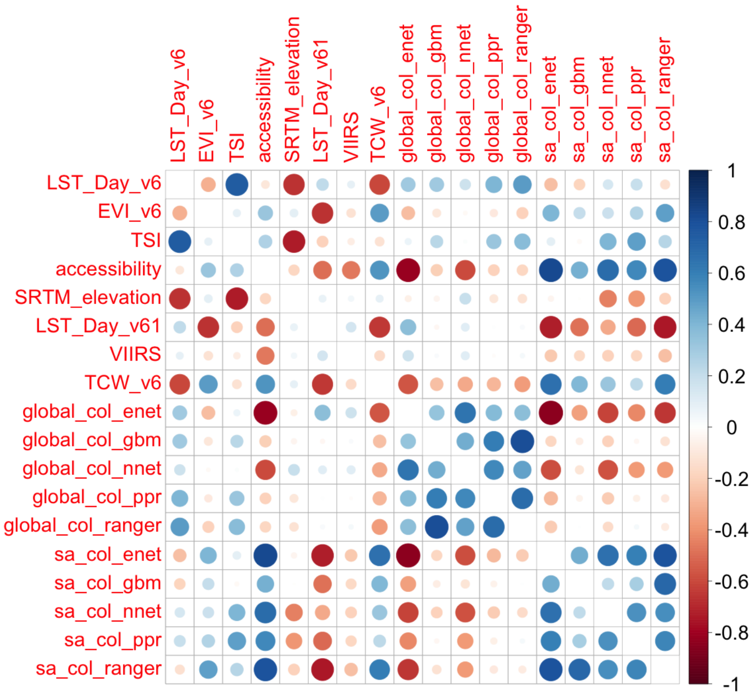

Figure S46: Correlation matrix for all covariates (raw environmental and machine learning predictions) for Colombia

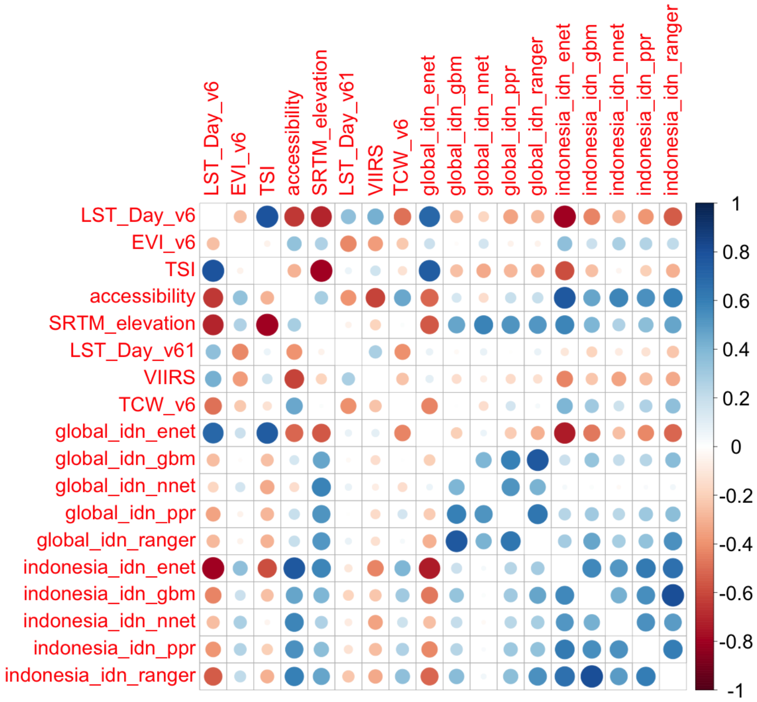

Figure S47: Correlation matrix for all covariates (raw environmental and machine learning predictions) for Indonesia

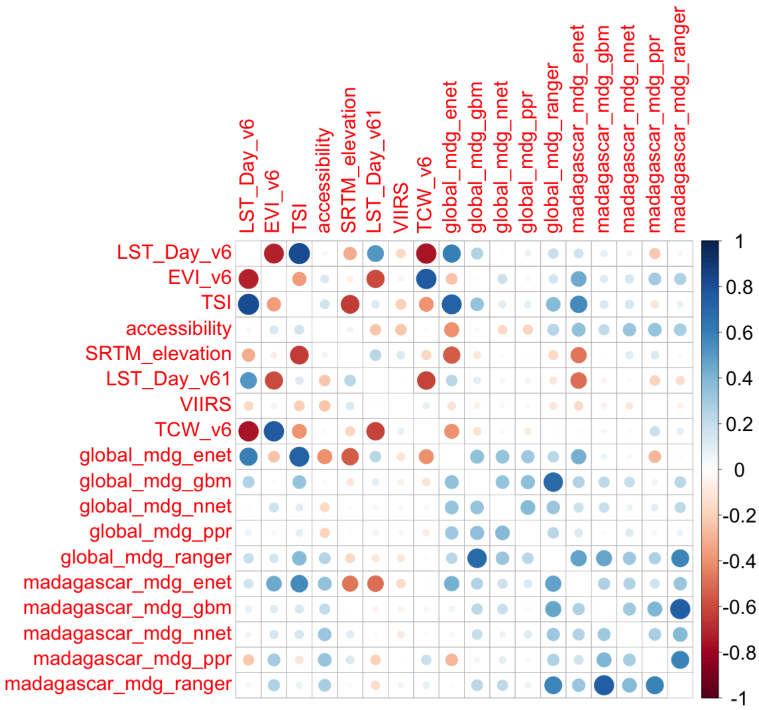

Figure S48: Correlation matrix for all covariates (raw environmental and machine learning predictions) for Madagascar

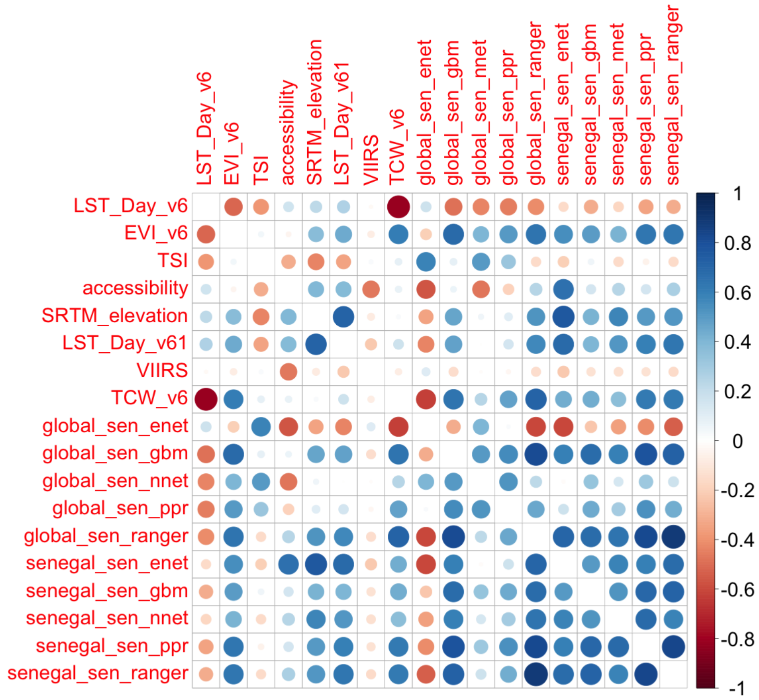

Figure S49: Correlation matrix for all covariates (raw environmental and machine learning predictions) for Senegal

8. Model consistency

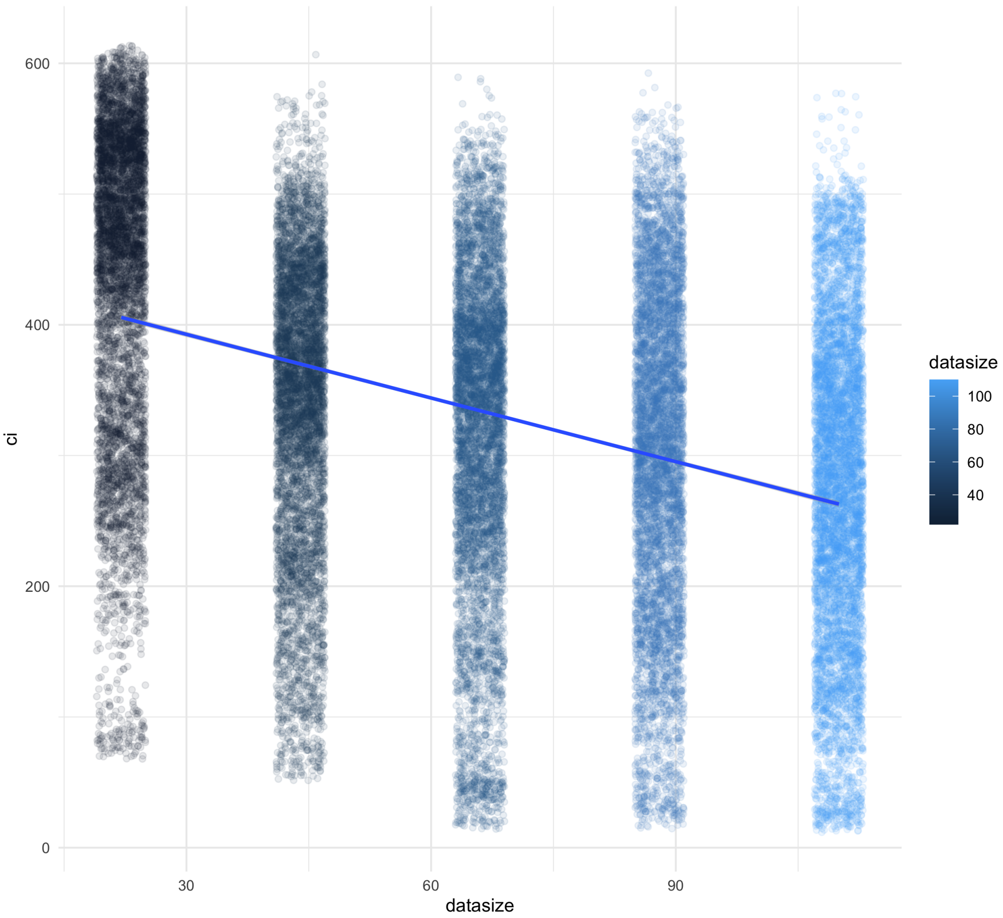

Figure S50: 80% credible interval widths for a some pixels when fitting models in Madagascar with increasing dataset sizes. The blue line is a simple linear model fit.
